# Geminin Overexpression Promotes Imatinib Sensitive Breast Cancer: A Novel Treatment Approach for Aggressive Breast Cancers, Including a Subset of Triple Negative

**DOI:** 10.1371/journal.pone.0095663

**Published:** 2014-04-30

**Authors:** Zannel Blanchard, Nicole Mullins, Pavani Ellipeddi, Janice M. Lage, Shawn McKinney, Rana El-Etriby, Xu Zhang, Raphael Isokpehi, Brenda Hernandez, Wael M. ElShamy

**Affiliations:** 1 Cancer Institute, University of Mississippi Medical Center, Jackson, Mississippi, United States of America; 2 Department of Biochemistry, University of Mississippi Medical Center, Jackson, Mississippi, United States of America; 3 Department of Pathology, University of Mississippi Medical Center, Jackson, Mississippi, United States of America; 4 Department of Surgery, University of Mississippi Medical Center, Jackson, Mississippi, United States of America; 5 Center of Biostatistics and Bioinformatics, University of Mississippi Medical Center, Jackson, Mississippi, United States of America; 6 Center for Bioinformatics & Computational Biology, Department of Biology, Jackson State University, Jackson, Mississippi, United States of America; 7 Cancer Research Center of Hawaii, University of Hawaii, Honolulu, Hawaii, United States of America; Dartmouth, United States of America

## Abstract

Breast cancer is the second leading cause of cancer-related deaths in women. Triple negative breast cancer (TNBC) is an aggressive subtype that affects 10–25% mostly African American women. TNBC has the poorest prognosis of all subtypes with rapid progression leading to mortality in younger patients. So far, there is no targeted treatment for TNBC. To that end, here we show that c-Abl is one of several tyrosine kinases that phosphorylate and activate geminin’s ability to promote TNBC. Analysis of >800 breast tumor samples showed that geminin is overexpressed in ∼50% of all tumors. Although c-Abl is overexpressed in ∼90% of all tumors, it is only nuclear in geminin overexpressing tumors. In geminin-negative tumors, c-Abl is only cytoplasmic. Inhibiting c-Abl expression or activity (using imatinib or nilotinib) prevented geminin Y150 phosphorylation, inactivated the protein, and most importantly converted overexpressed geminin from an oncogene to an apoptosis inducer. In pre-clinical orthotopic breast tumor models, geminin-overexpressing cells developed aneuploid and invasive tumors, which were suppressed when c-Abl expression was blocked. Moreover, established geminin overexpressing orthotopic tumors regressed when treated with imatinib or nilotinib. Our studies support imatinib/nilotonib as a novel treatment option for patients with aggressive breast cancer (including a subset of TNBCs)-overexpressing geminin and nuclear c-Abl.

## Introduction

Licensing at origin of replications (ORI) starts by sequential binding of component of the pre-replication complex (pre-RC); origin recognition complex (ORC), Cdc6 and Cdt1 [1], [2] followed by binding of the rate limiting complex; the mini-chromosome maintenance complex (MCM2-7) [1], [2]. Geminin, a coiled-coil protein without sequence homology to any known protein [3] inhibits re-replication by binding to Cdt1 at ORI and preventing MCM binding, thus, maintaining chromosomal integrity and number [1], [2]. However, other critical roles for geminin have recently been identified. For instance, in *Drosophila*, geminin regulates neural cell fate determination, and in mouse it regulates cellular differentiation by binding to and antagonizing Six3 or Hox transcriptional activity [4]–[7].

More recently, we showed that geminin is recruited to chromatin (also centrosomes, centromeres and midbody) from the nuclear soluble fraction at the end of S-phase after being tyrosine phosphorylated to promote proper cytokinesis and M-to-G_1_ transition [8]–[10]. Geminin accomplishes that by binding to and activating, at least two important cytokinesis inducers; namely topoisomerase II alpha (TopoIIα) [9] and Aurora B kinase (AurB) [10]. However, when overexpressed geminin prematurely inhibits chromosomal decatenation by inactivating TopoIIα [9] and condensation/segregation by inactivating AurB, and inhibiting histone H3(S10) phosphorylation (p-H3^S10^) [10], [11], which triggers aneuploidy.

Interestingly, previously we showed that overexpression of any single tyrosine mutant geminin (human geminin carries 3 tyrosine residues, at positions 98, 111 and 150) has no effect on H3^S10^ phosphorylation, chromosome decatenation/condensation/segregation and triggers apoptosis instead of aneuploidy [9], [10]. Suggesting that geminin is a genuine oncogene that promotes genomic instability when overexpressed as a wild-type protein. In keeping with that, geminin is overexpressed in many tumor types [12]–[16], and to our knowledge the protein is wild type in these tumors. In fact, we recently analyzed a cohort of 150 DNA samples freshly isolated from patients’ breast tumors and found that geminin gene carries no mutation or any alterations that can affect the protein in all these tumors (ElShamy WM and Iglehart D, unpublished data). The proto-oncogene c-Abl is a tightly regulated, ubiquitously expressed multifunctional non-receptor protein tyrosine kinase [17]–[21]. c-Abl can be localized to the plasma membrane, cytoplasm and nucleus and affects a variety of cellular functions and activities. For example, cytoplasmic c-Abl plays an important role in cell proliferation, differentiation, and migration [22], [23], whereas nuclear c-Abl induces apoptosis, DNA damage repair [22], [23] or chromatin dynamic by phosphorylating heterochromatic histones [24]. Unlike chronic myelogenous leukemia [CML] [23], in solid tumors, such as glioblastoma, melanoma, non-small-cell lung cancer, breast and gastric carcinomas there are no c-Abl translocations. Instead, in these solid tumors, c-Abl is overexpressed [24]–[27]. Imatinib mesylate (*aka* Gleevec or STI571) [28], is a small molecule inhibitor that targets the ATP-binding site in c-Abl kinase domain and is successful in treating CML patients [29], [30], as well as gastrointestinal stromal tumors (GIST) expressing mutant c-KIT, or overexpressing α- or β platelet-derived growth factor receptors (PDGFRα or β) [31]. Nilotinib [*aka* Tasigna] is a novel tyrosine kinase inhibitor that inhibits BCR-ABL [30]–[35], c-KIT and PDGFRα approved to treat CML or GIST patients, especially those showing imatinib-resistance or -intolerance [30]–[33].

Here, we expand our previous data and show that c-Abl binds directly or indirectly to geminin and phosphorylates Y150 in G_2_/M/early G_1_ cells. Inhibiting Y150 phosphorylation by suppressing c-Abl expression or activity destabilizes endogenous as well as exogenous geminin proteins, prevents aneuploidy and triggers cell death, specifically, in geminin overexpressing cells. c-Abl is exclusively nuclear in human breast tumors overexpressing geminin (such as TNBCs). Interestingly, orthotopic tumors developed in mice using geminin overexpressing cells also showed exclusive nuclear c-Abl expression. c-Abl silencing or imatinib treatment effectively kills geminin-overexpressing tumors in this pre-clinical model. We propose imatinib/nilotinib as a novel treatment option for patients with aggressive breast cancer (including a subset of TNBCs)-overexpressing geminin and nuclear c-Abl.

## Results

### c-Abl binds and phosphorylates geminin in G_2_/M cells

**Figure 1:**
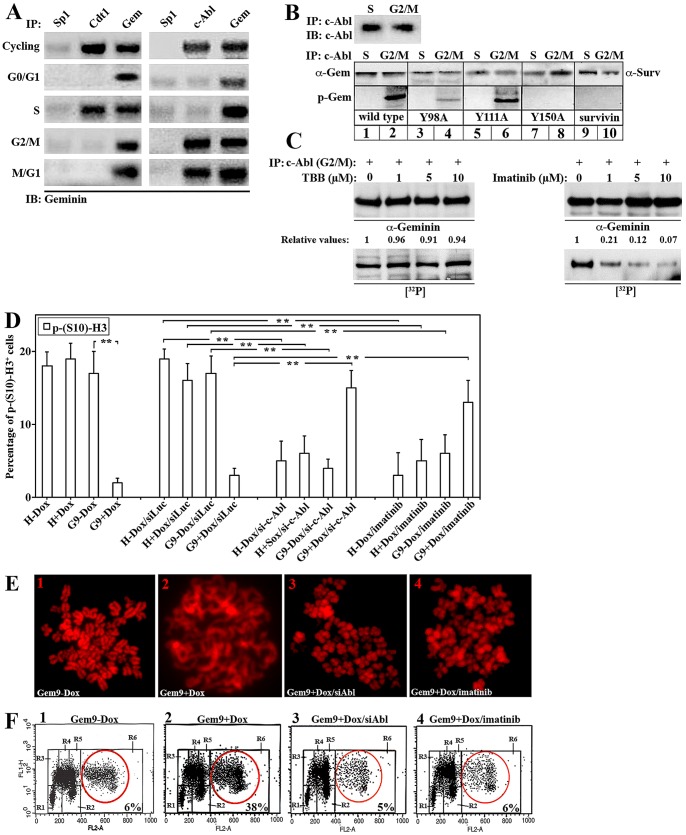
c-Abl binding and phosphorylation of geminin Y150 in G_2_/M/early G_1_ cells promotes overexpressed geminin oncogenicity in HME cells. (A) Immunoprecipitation of cycling, G_0_/G_1_, S, G_2_/M or M/G_1_ HME cells with anti-Cdt1, -c-Abl, -Sp1 (negative control) and -geminin antibodies. (B) c-Abl immunoprecipitated from S or G_2_/M HME cells (upper panels) was used to *in vitro* phosphorylate GST-WT-, -Y98A-, -Y111A-, -Y150A-geminin or GST-survivin (negative control, lower panels). (C) c-Abl immunoprecipitated from G_2_/M was used to *in vitro* phosphorylate GST-WT-geminin in the presence of the increasing concentrations of CKII inhibitor, TBB (left panels) or c-Abl inhibitor, imatinib (right panels). (D) The percentage of p-(S10)-H3^+^-cells in HME, uninduced or induced Gem9 following transfection of si-control or si-c-Abl (for 72 hr) or treatment with vehicle or 5 µM of imatinib (during the last 24 h). Data are represented as mean ± SD of triplicates done three separate times, where *  =  *p*≤*0.05* and **  =  *p*≤*0.001*. (E) Metaphase spread analysis of chromosome condensation in uninduced or induced Gem9 cells before or after transfection of sic-Abl or treatment with 5 µM of imatinib. (F) FACS analysis of uninduced or induced Gem9 cells transfection of sic-Abl or treatment with 5 µM of imatinib. Aneuploid cells are shown in the red circles and their percentage is in insets. R1 = G_0_/G_1_, R3/R4/R5 = early/mid/late S, R2 = G_2_/M and R6 = >4N cells. Experiments were done three separate times in triplicates.

Kinases often bind their targets. To identify kinases that phosphorylate geminin and induce its G_2_/M function, sonicated extracts (to isolate all cellular proteins) from S- or G_2_/M-synchronized human mammary epithelial (HME) cells (for efficiency of synchronization protocol, see Fig. S1A–D) were immunoprecipitated (IPd) using a mono-specific anti-geminin antibody. Co-IPd proteins (see Fig. S1E) were then identified using micro-sequencing technique. Geminin antibody co-IPd its S-phase partner, Cdt1 [2] only from S-phase cells (validating the approach). In this assay, we found that the non-receptor tyrosine kinase, c-Abl was IPd using anti-geminin antibody only from G_2_/M cells. To confirm these results, sonicated cycling or G_0_/G_1_-, S-, G_2_/M- and M/G_1_-synchronized HME cells were IPd with anti-Cdt1, anti-c-Abl, anti-geminin or anti-Sp1 (as negative control) antibodies followed by immunoblotting with anti-geminin antibody. Again, Cdt1 antibody pulled down geminin from S-phase extract only (Fig. 1A, left) and c-Abl antibody pulled down geminin from G_2_/M and M/G_1_ extracts (Fig. 1A, right).

To study whether geminin is a substrate for c-Abl, beads bound c-Abl IPd from sonicated S- or G_2_/M-synchronized HME cells extracts (note equal amount of c-Abl IPd from each phase, Figure 1B, upper panel) were used to *in vitro* phosphorylate WT or different tyrosine mutant geminin. First, c-Abl isolated from S-phase cells failed to phosphorylate any of the proteins used (Fig. 1B, lower panel). Second, survivin seemed not to be a substrate of c-Abl isolated from G_2_/M cells, at least in this assay (see Fig. 1B, lower panel lanes 9 and 10). On the other hand, wild type (WT)-geminin is a substrate of c-Abl isolated G_2_/M cells (compare lane 2 to 1 in Fig. 1B, lower panel). Moreover, c-Abl isolated from G_2_/M cells also phosphorylated Y98A-geminin (compare lane 4 to 3 in Fig. 1B, lower panel) and Y111A-geminin (compare lane 6 to 5 in Fig. 1B, lower panel) but failed to phosphorylate Y150A-geminin (Fig. 1B, lower panel lanes 7 and 8).

To further confirm that, beads-bound c-Abl isolated from G_2_/M HME cells was incubated with WT-geminin in the presence of increasing concentrations of 4,5,6,7-tetrabromobenzotriazole (TBB, a CKII specific inhibitor, 35) or imatinib. As expected, imatinib and not TBB significantly decreased the phosphorylation of WT-geminin by G_2_/M-phase c-Abl (Figure 1C, right lower panels). Taken together, suggests that c-Abl binds and phosphorylates geminin on Y150 in G_2_/M/early G_1_ phases.

### c-Abl silencing/inactivation restores histone H3^S10^ phosphorylation, chromosome condensation and suppresses aneuploidy in geminin overexpressing cells

We recently showed that WT geminin overexpression leads to diminution of H3^S10^ phosphorylation (p-H3^S10^), which leads to lack of chromosome condensation/segregation, which in turn leads to aneuploidy in HME cells [10]. In contrast, overexpression of Y150A-geminin did not prevent p-H3^S10^ and chromosome condensation/segregation and did not induce aneuploidy in HME cells [10].

To study whether inhibiting Y150 phosphorylation will reverse geminin overexpression effect on H3^S10^ phosphorylation, we compared p-H3^S10^ levels in uninduced Gem9 [a HME cell clone containing a doxycycline (Dox)-inducible geminin allele, analysis of a second clone #10 gave essentially identical results, and thus for simplicity only data with clone # 9 are presented] see [9] and [10] cells, induced Gem9 cells (by incubation with 2 µg/ml of Dox for 72 h), induced Gem9 cells (72 h) silenced from c-Abl (72 h, see siRNA specificity in Fig. S1F, left panels) or induced Gem9 cells (72 h) treated with 5 µM imatinib (for 24 h, see the reduction in activity as depicted by the reduction in the phosphorylation of the c-Abl direct target, CrkII [36] without affecting expression of c-Abl, Fig. S2A, left panels). Naïve HME cells were treated in the same manner as a negative control. On the day of the experiment, all samples were labeled with FITC-labeled anti-p-H3^S10^ antibody and processed with FACS. As expected, geminin overexpression blocked p-H3^S10^ (Fig. 1D) and blocking c-Abl expression or activity almost completely restored p-H3^S10^ in induced Gem9 cells (Fig. 1D).

To study whether inhibiting Y150 phosphorylation will reverse geminin overexpression effect on chromosome condensation [10], we compared chromosome condensation in uninduced Gem9 cells, induced Gem9 cells (7 days), induced Gem9 cells silenced from c-Abl (7 day, siRNA transfected every 3^rd^ day) and induced Gem9 cells treated with 5 µM imatinib (7 days, drug added daily). On the day of the experiment, all cultures were exposed to 10 µM of the microtubules depolymerizing agent; colcemid [37] for 1 h before metaphase chromosome spread was performed. As expected, colcemid triggered chromosome condensation in the uninduced Gem9 cells [10], but failed to do so in induced Gem9 cells (compare 1 to 2 in Fig. 1E). Impressively, c-Abl silencing (3 in Fig. 1E) or inactivation (4 in Fig. 1D) almost completely restored chromosome condensation in induced Gem9 cells.

To study whether inhibiting Y150 phosphorylation will reverse geminin overexpression-induced aneuploidy, we compared aneuploidy in uninduced Gem9, induced Gem9 cells (72 h), induced Gem9 cells silenced from c-Abl (for 72 h) and induced Gem9 cells treated with 5 µM of imatinib (72 h, drug added daily). On the day of the experiment, samples were switched to medium containing 2 µM BrdU (for an additional 24 h) before they were collected, labeled with PI and FITC-anti-BrdU antibody and analyzed with FACS. Unlike uninduced Gem9 culture, induced Gem9 culture showed high percentage of cells with >4N DNA content (38% vs. 6%, compare red circle in Fig. 1F/2 to 1F/1). Again, c-Abl silencing or inactivation significantly decreased aneuploidy induced by geminin overexpression (5%, and 6%, respectively, see red circles in 3 and 4 in Fig. 1F). Taken together, these data clearly show that geminin phosphorylated on Y150 by c-Abl acts as an oncogene that induces aneuploidy when overexpressed by preventing H3^S10^ phosphorylation and chromosome condensation/segregation.

### c-Abl silencing/inactivation triggers apoptosis, specifically in geminin overexpressing cells

**Figure 2:**
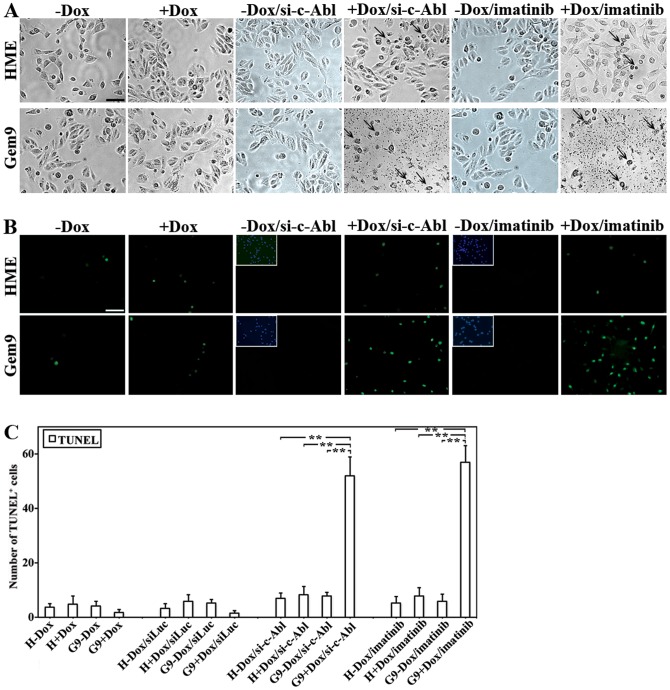
c-Abl silencing or inactivation promotes cell death, specifically, in geminin overexpressing cells and prevents transformation. (A) Phase contrast images showing naïve HME, uninduced and induced Gem9 cultures following transfection of sic-Abl or treatment with 10 µM of imatinib. Scale bar  =  50 µm. (B) Representative images showing TUNEL^+^-cells in naïve HME, uninduced and induced Gem9 cultures following transfection of sic-Abl or treatment with 10 µM of imatinib. Inset is DAPI stained cells in the corresponding images. Scale bar  =  100 µm. (C) Number of TUNEL^+^-cells in naïve HME, uninduced and induced Gem9 cultures after c-Abl silencing or inactivation with imatinib. Data are represented as mean ± SD of triplicates done 3 separate times, where **  =  *p*≤*0.01* and ***  =  *p*≤*0.0001*.

Because overexpression of Y150A-geminin triggered apoptosis instead of aneuploidy see [10], we wondered whether inhibiting Y150 phosphorylation will have the same effect. We compared cell death in uninduced Gem9 cells, induced Gem9 cells (72 h for TUNEL and 96 h for morphological analysis), induced Gem9 cells silenced from c-Abl (72 h for TUNEL and 96 h for morphological analysis) or induced Gem9 cells (72 for TUNEL and 96 h for morphological analysis) treated with 10 µM imatinib (24 h for TUNEL and 48 h for morphological analysis). On the day of the experiment, cells were observed under light microscope and photographed or processed for TUNEL analysis, photographed and counted. Silencing or inactivation of c-Abl had no effect on the survival of naïve HME or uninduced Gem9 cells as detected by the lack of morphologically (Fig. 2A) or TUNEL^+^ (Fig. 2B and 2C) dying cells. In contrast, c-Abl silencing or inactivation triggered significant number of morphologically (see arrows in Fig. 2A) as well as TUNEL^+^ (Fig. 2B and 2C) dying cells in induced Gem9. Furthermore, c-Abl silencing or inactivation also triggered apoptosis in MDA-MB-231 cells (endogenously overexpressing geminin) as detected by the increase in sub-G1 fraction (compared B and C to A in Fig. S3).

**Figure 3:**
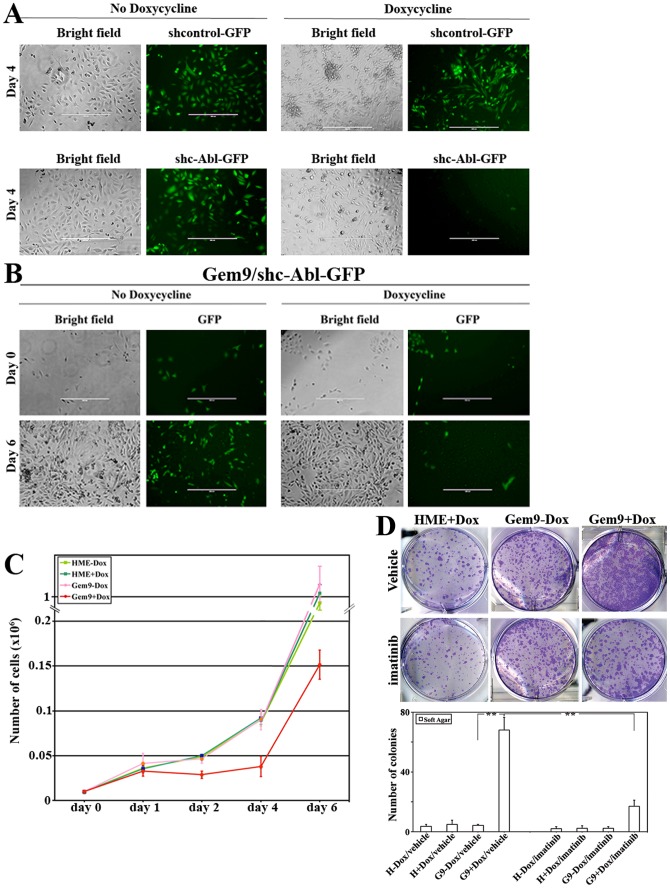
Death of geminin overexpressing cells specifically in the absence of c-Abl. (A) Representative bright field and fluorescence images showing naïve HME, uninduced and induced Gem9 cells transfected with sh-control or shc-Abl and grown in the presence or absence of doxycycline for 4 days. Scale bar  =  400 µm. (**B**) Representative bright-filed and fluorescence images of Gem 9 cells expressing sh-control or shc-Abl and grown in the presence or absence of doxycycline for 0 or 6 days. Scale bar  =  400 µm. (C) Quantitative analysis of the data in (A) and (B). (D) Phase contrast images showing colony formed in soft agar using naïve HME, uninduced and induced Gem9 cultures before or after treatment with 10 µM of imatinib (upper) Quantitative analysis of the soft agar experiment described in (lower). Data are represented as mean ± SD from triplicates done 3 separate times. ***  =  *p*<*0.001*.

To confirm these data further, naïve HME and Gem9 cells were transfected with constructs expressing sh-control/GFP or sh-c-Abl/GFP (see depletion efficiency using this sh-c-Abl in Fig. S1F, right panels). Unselected cultures were then incubated or not with Dox for 6 days. Cells were detected in all cultures using bright field objective. However, the same fields contained GFP^+^ cells (detected using fluorescence objective) only in cultures of naïve HME ± Dox and uninduced Gem9 cells (Fig. 3A). To follow the kinetics of cell death induced by c-Abl silencing in these geminin-overexpressing cells, Gem9 cells expressing sh-c-Abl/GFP were grown ± Dox for 0, 1, 2, 4 or 6 days and GFP^+^ cells were photographed and counted daily in 10 high magnification fields. While similar numbers of GFP^+^ cells were found at day 0 and 1 in all cultures (Fig. 3B and C), the number of GFP^+^ cells began to gradually decrease in induced Gem9 cells compared to uninduced Gem9 cells starting at day 2 (Fig. 3B and C). By day 6 compared to uninduced Gem9 cells, induced Gem9 cells contained only 10% of GFP^+^ cells (Fig. 3C). Taken together, these data clearly show that c-Abl silencing or inactivation triggers death of geminin overexpressing cells, specifically.

### c-Abl silencing/inactivation suppresses geminin overexpression induced transformation

Unlike WT-geminin, Y150A-geminin overexpression did not transform HME cells (10). Whether inhibiting Y150 phosphorylation will have the same effect was studied next. Naïve HME and Gem9 cells grown for 72 h ± Dox were re-plated on agar containing wells ± Dox and 10 µM imatinib for 14 days. On the day of the experiment, colonies formed under each condition were photographed and counted. Unlike naïve HME or uninduced Gem9 cells that formed few small colonies (Fig. 3D, upper), induced Gem9 cells formed many large colonies (Fig. 3D, upper). Imatinib had no effect on naïve HME or uninduced Gem9 cells, but significantly reduced the number and size of colonies formed by induced Gem9 cells (Fig. 3D, upper). Quantitative analysis of this experiment is presented in Fig. 3D (lower). Taken together, c-Abl silencing or inactivation suppresses geminin overexpression-induced transformation, most likely because it induces cell death specifically in these cells.

### Y150 phosphorylation by c-Abl stabilizes geminin

**Figure 4:**
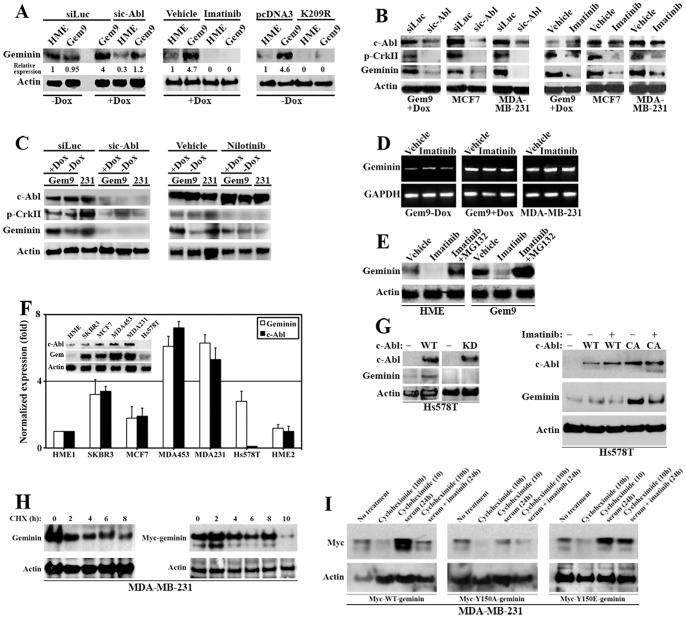
Stabilization of geminin protein by c-Abl phosphorylation and the expression of geminin and c-Abl in breast cancer cell lines. (A) The expression level of geminin in naïve HME, uninduced and induced Gem9 cells following c-Abl silencing or inactivation using imatinib or transfection of the dominant negative c-Abl (K290R). (B) The expression of c-Abl, geminin, p-CrkII in induced Gem9, MCF7 or MDA-MB-231 cells silenced from c-Abl or treated with imatinib. (C) The expression level of c-Abl, p-CrkII and geminin in induced Gem9 or MDA-MB-231 cells silenced of c-Abl or treated with nilotinib. (D) RT/PCR analysis of geminin mRNA in uninduced, induced Gem9 or MDA-MB-231 cells in the presence of vehicle or imatinib. (E) The expression of geminin in naïve HME or induced Gem9 cells in the presence of vehicle, imatinib or imatinib + MG132. (F) The expression of c-Abl and geminin mRNAs and proteins (inset) in several breast cancer cell lines. Note that Hs578T cells express high level of geminin mRNA, but no protein. (G) The re-expression of geminin in Hs578T cells reconstituted with WT and not kinase dead (KD) c-Abl (left). The re-expression of geminin in Hs578T cells reconstituted with WT or constitutively active (CA) c-Abl was blocked by imatinib (right). (H) The expression of endogenous (left) or overexpressed (right) geminin in MDA-MB-231 cells following treatment with the translational inhibitor cycloheximide (CHX) for 0-10 h with cells collected at 2 h intervals. (I) The expression of exogenous Myc tagged WT- (left), Y150A- (middle) or Y150E- (right)-geminin following no treatment (1^st^ lanes), 10 h of CHX (2^nd^ lanes), 10 h CHX followed by 24 h of complete serum (3^rd^ lanes) or 10 h CHX followed by 24 h of complete serum + 10 µM of imatinib (4^th^ lanes).

We aimed next to understand the mechanism involved in the induction of apoptosis in geminin overexpressing cells following c-Abl silencing or inactivation. We compared the expression of geminin in HME and uninduced Gem9 cells, induced Gem9 cells (72 h), induced Gem9 cells silenced from c-Abl (72 h), induced Gem9 cells (72 h) transfected with a dominant negative c-Abl construct (i.e. K209R) [26] (for 48 h) or induced Gem9 cells (72 h) treated with 10 µM imatinib (24 h). Sonicated extracts from all cultures were probed for geminin. A significant reduction in endogenous (i.e. in naïve HME) as well as overexpressed (i.e. in induced Gem9) geminin protein levels was detected following c-Abl silencing or inactivation (Fig. 4A). The same was also true in two breast cancer cell lines, endogenously overexpressing geminin, namely MCF7 and MDA-MB-231 cells (Fig. 4B), which was correlated with significant reduction in the phosphorylation of c-Abl downstream target; CrkII (Fig. 4B). Further analysis showed that no detectable levels of c-Kit or PDGFR (two of imatinib known targets) [32] could be measured in naïve HME, uninduced or induced Gem 9 cells (not shown). Indeed, similar results were obtained in the presence of nilotinib (another c-Abl inhibitor). Like silencing, c-Abl inactivation using imatinib or nilotinib significantly decreased p-CrkII levels and geminin expression in uninduced Gem9, induced Gem9 and MDA-MB-231 cells (Fig. 4C). Taken together, the data so far support the view that in HME or induced Gem9 cells imatinib/nilotinib effects are exerted predominantly upon c-Abl, although we cannot exclude other c-Abl targets involvement in this process.

To rule out an effect of c-Abl silencing or inactivation on geminin mRNA expression/stability, c-Abl was inactivated with 10 µM of imatinib in uninduced, induced Gem9 or MDA-MB-231 cells, mRNAs were isolated and the expression of geminin mRNA in these cells was analyzed using RT/PCR. Imatinib treatment had no effect on the expression of geminin mRNA in any of the cell lines (Fig. 4D), reinforcing the fact that c-Abl inactivation does not affect geminin transcription (i.e. in naïve HME and MDA-MB-231) or geminin mRNA stability (i.e. in induced Gem9 cells, since in these cells transcription ensues from heterologous promoter, Fig. 4C) but mainly exerted on the geminin protein stability.

Finally, to clearly show that Y150 phosphorylation by c-Abl stabilizes geminin protein, naïve HME or induced Gem9 (72 h) were grown in the presence of vehicle, 10 µM imatinib or 10 µM imatinib+10 µM MG132 (proteasome inhibitor) for an extra 24 h. Using sonicated extracts we showed that imatinib significantly decreased the level of geminin in naïve HME and induced Gem9 cells (Fig. 4E) and that MG132 treatment blocked that effect (Fig. 4E). Taken together, these data show that Y150 phosphorylation by c-Abl stabilizes geminin protein and that preventing Y150 phosphorylation promotes cell death only in geminin overexpressing cells, perhaps because these cells are addicted to some or all of geminin oncogenic functions.

### Geminin and c-Abl mRNAs and proteins are co-overexpressed in breast cancer cell lines

To pursue this hypothesis further we searched for cell line endogenously lacking c-Abl expression. Compared to naïve HME cell lines (HME1 and HME2, Fig. 4F), all breast cancer cell lines tested showed high expression level of c-Abl and geminin mRNAs (Fig. 4F) and proteins (inset in Fig. 4F). One cell line; Hs578T, while showed high expression of geminin mRNA and lacked c-Abl mRNA (Fig. 4F), it lacked expression of both proteins. This reinforces our above mentioned conclusion that despite the overexpression of geminin mRNA, in Hs578T cells the lack of c-Abl expression either blocked translation of geminin mRNA or promoted degradation of geminin protein. Therefore, Hs578T cells could be the perfect cell line to study this connection in depth.

Hs578T cells were transfected with wild type (WT), kinase dead (KD) or constitutively active (CA) c-Abl cDNA. Although all variants were equally expressed 48 h post-transfection (Fig. 4G), geminin was only re-expressed in cells transfected with WT or CA c-Abl (Fig. 4G). More importantly, treating these transfected cells with 10 µM imatinib blocked this re-expression (Fig. 4G, right). This suggests that only active c-Abl that can phosphorylate geminin Y150 promotes re-expression of geminin protein in Hs578T cells. In keeping with this conclusion, only Myc-tagged 3Y-to-E-geminin (i.e. all tyrosine residues were replaced with glutamic acids, i.e. constitutively active geminin) and not Myc-WT- or Myc-3Y-to-A-geminin was expressed when transfected in Hs578T cells (Fig. S2B).

To establish that even further and to show that Y150 is indeed the target for c-Abl, we developed an affinity purified rabbit polyclonal anti-p-Y150-geminin antibody. Hs578T cells transfected with a WT or CA c-Abl cDNA (48 h) were treated with vehicle or 10 µM of imatinib for an additional 24 h. Sonicated extracts were then probed with anti-geminin antibodies that recognize total geminin or p-Y150-geminin. As above, WT or CA c-Abl overexpression triggered geminin re-expression in Hs578T (Fig. S2C) that was phosphorylated on Y150 (Fig. S2C). Imatinib treatment significantly blocked this re-expression of total (Fig. S2C) and more importantly of p-Y150 (Fig. S2C) geminin in these cells. Using ImageJ software (NIH), we showed that imatinib reduced re-expression of total geminin by ∼30% and p-Y150 geminin by ∼60% compared to actin in cells transfected with WT c-Abl and total geminin by ∼50% and p-Y150 geminin by ∼80% compared to actin in cells transfected with CA c-Abl (Fig. S2C, lower). Additionally, only ∼60% or ∼40% of total geminin remained following imatinib treatment in cells transfected with WT- or CA c-Abl cDNAs were Y150 phosphorylated, respectively (Fig. S2C, lower). This was also true in MDA-MB-231 cells transfected with si-c-Abl (72 h, Fig. S2D).

Finally, to measure geminin half-life, MDA-MB-231 were transfected with Myc-WT-geminin. Forty-eight hours later, cells were switched to medium containing 10 µM of the protein synthesis inhibitor; cycloheximide (CHX). At 2, 4, 6, 8 or 10 h following CHX treatment, cells were collected, sonicated and then probed for endogenous geminin half-life using anti-geminin antibody or exogenous geminin half-life using anti-Myc tag (9E10) antibody. A gradual decrease in endogenous (Fig. 4H, left and Fig. S2E) and exogenous (see Fig. 4H, right, and Fig. S2E) geminin was observed. After ∼10 h most of cellular geminin (endogenous or exogenous) were completely abolished (Fig. 4H, left and Fig. S2E).

Having established geminin half-life, we next transfected MDA-MB-231 cells with Myc-WT-, -Y150A- or -Y150E-geminin. Forty-eight hours later all cultures were switched to media containing 10 µM of CHX, which was replaced 10 h later with media containing 10 µM of vehicle or imatinib for 24 h. All cultures were then sonicated and probed for Myc-geminin using anti-Myc tag antibody. All constructs were expressed at almost equivalent levels (1^st^ lanes in all panels in Fig. 4I). After 10 h in CHX, almost complete abolishment of all variants was detected (2^nd^ lanes in all panels in Fig. 4I). As expected, washing the CHX away promoted expression of WT- and Y150E- but had little effect on Y150A-geminin (3^rd^ lanes in all panels in Fig. 4I). Impressively, imatinib treatment prevented re-expression of WT-geminin, but largely had no effect on the re-expression of Y150E-geminin protein (4^th^ lanes in all panels in Fig 4I). Taken together, these data clearly show that only active c-Abl phosphorylates Y150 and stabilizes geminin protein.

### Geminin overexpression promotes aggressive breast tumors that co-overexpress nuclear c-Abl only

**Figure 5:**
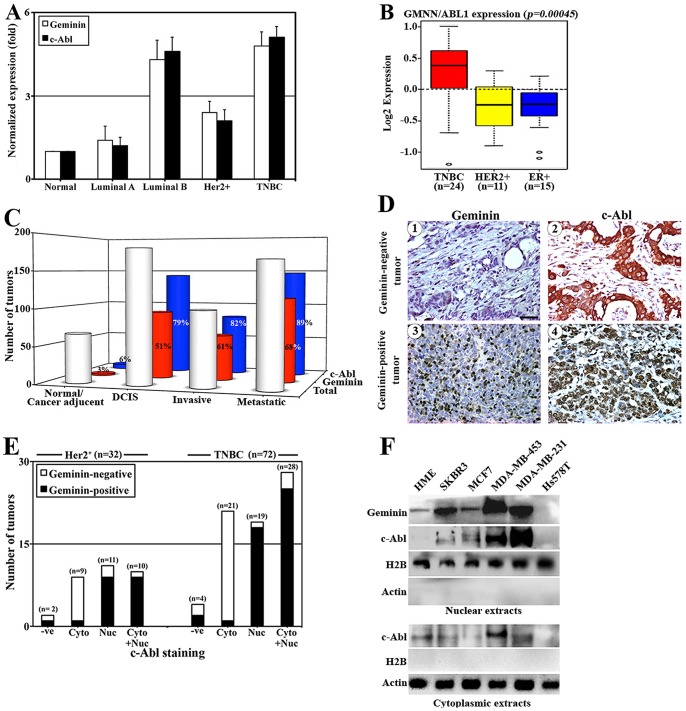
The expression of geminin and c-Abl in breast tumor samples. (A) The normalized expression of geminin and c-Abl mRNA in normal (n = 5), luminal A (n = 7), luminal B (n = 9), Her2^+^ (n = 11) and TN/BL (n = 7) tumor samples. (B) Box plot of gene expression for combined gene set of geminin and c-Abl across cell lines grouped into clinical subtypes; triple negative breast cancer (TNBC, red), HER2-positive (HER2, yellow), and ER-positive (ER^+^, blue) based on annotation data from (38). (C) Number of total, geminin-positive and c-Abl-positive tumors detected using immunohistochemistry on normal/cancer adjacent (n = 66), DCIS (n = 180), invasive (n = 100) and metastatic (n = 165) breast tumors. (D) Representative immunohistochemical staining images of geminin (1 and 3) or -c-Abl (2 and 4) on invasive breast tumor samples. Scale bar  =  50 µm. (E) Number of geminin-positive or -negative in Her2^+^ (n = 32) or TN/BL (n = 72) showing cytoplasmic (Cyt), nuclear (Nuc), or both (Cyt + Nuc) c-Abl expression. (F) The level of geminin and c-Abl in the nuclear or cytoplasmic fractions of the indicated cell lines.

To analyze geminin and c-Abl expression in primary breast tumor samples, we first performed real time RT/PCR on a cohort of breast tumors of different subtypes. Geminin and c-Abl mRNAs levels are low in normal tissue (n = 5) and luminal A tumors (n = 7), moderate in Her2^+^ tumors (n = 11), and high in luminal B (n = 9) and triple negative breast cancer (TNBC) [38], [39] (n = 7) tumors (Fig. 5A). In fact, using the GSA-cell line application in Gene Expression-Based Outcome (GOBO) for Breast Cancer Online, we found that among a panel of 50 breast cancer cell lines grouped according to clincal subtypes [40], TNBC cell lines displayed the highest merged geminin and c-Abl mRNA expression (Fig. 5B).

Next, two cohort of paraffin embedded tissue microarrays (TMAs), the first a training cohort and the second a conformational cohort were immunohistochemically (IHC) stained with anti-geminin and -c-Abl antibodies. In the training cohort, geminin positive staining was detected in 3% of normal tissue, 51% of DCIS tumors, 61% of invasive tumors and 68% of metastatic tumors (Fig. 5C). C-Abl staining was detected in 6% of normal tissue, 79% of DCIS tumors, 82% of invasive tumors, and 89% of metastatic tumors (Fig. 5C). Interestingly, geminin-negative tumors expressed exclusively cytoplasmic c-Abl (see example of invasive tumor in Fig. 5D/1 and 2), whereas geminin-positive tumors expressed exclusively nuclear c-Abl (see example of invasive tumors in Fig. 5D/3 and 4).

In the conformational cohort, although 91% of the tumors were c-Abl-positive, and only 52% of the tumors were geminin-positive, 57% of the c-Abl-positive tumors expressed nuclear c-Abl and were geminin-positive, whereas the other 43% expressed cytoplasmic c-Abl and were geminin-negative (Table S1). State v.11 Fisher’s exact test confirmed a significant association between geminin and nuclear c-Abl (*p-value* = 0.0006, Table S1), and spearman correlation coefficient test also confirmed the highly significant correlation between expression of geminin and nuclear c-Abl with r  =  0.5432 (*p* = 0.0001).

Two cohorts of HER2^+^ (n = 32) and TNBC (n = 72) were identified and re-analyzed for the expression of geminin and nuclear vs. cytoplasmic c-Abl. In both cohorts, the majority if not all of the geminin-negative tumors exclusively expressed cytoplasmic c-Abl (Fig. 5E), whereas geminin-positive tumors expressed nuclear or mostly nuclear with some cytoplasmic c-Abl staining (Fig. 5E). Taken together these data show that geminin overexpressing tumors co-overexpress nuclear and not cytoplasmic c-Abl. Similar association was also detected in breast cancer cell lines, where we found that cell lines expressing the highest levels of geminin e.g., MDA-MB-231 and MDA-MB-453 showed predominantly nuclear c-Abl expression (Fig. 5F), whereas cell lines expressing low levels of geminin e.g., MCF7 and SKBR3 showed predominantly cytoplasmic c-Abl expression (Fig. 5F).

### Geminin/c-Abl co-overexpression is associated with worst outcome

To study next the association between geminin and/or c-Abl expression and disease outcome, we again re-analyzed the two cohorts studied earlier. All HER2^+^/geminin-negative tumors were grade 2 (G2, *p* = 0.0723). The majority were localized tumors, whereas few showed lymph node (LN)-positivity (*p* = 0.0503, Table S2). In contrast, few HER2^+^/geminin-positive tumors were G2 and the majority were G3 tumors (*p* = 0.0012). Few of these were localized tumors, whereas the majority showed either LN-positivity or distant-metastases (*p* = 0.0109, Table S2). Similarly, in the TNBC/geminin-negative tumors, the majority of the tumors were G2, while few were G3 tumors (*p* = 0.0525). The majority of these were localized tumors and few showed LN-positivity (*p* = 0.0042, Table S2). In contrast, in TNBC/geminin-positive tumors, few were G2 tumors, whereas the majority were G3 tumors (*p* = 0.0056). Few of these tumors were localized, whereas the majority showed LN-positivity or distant-metastases (*p* = 0.0022, Table S2).

**Figure 6:**
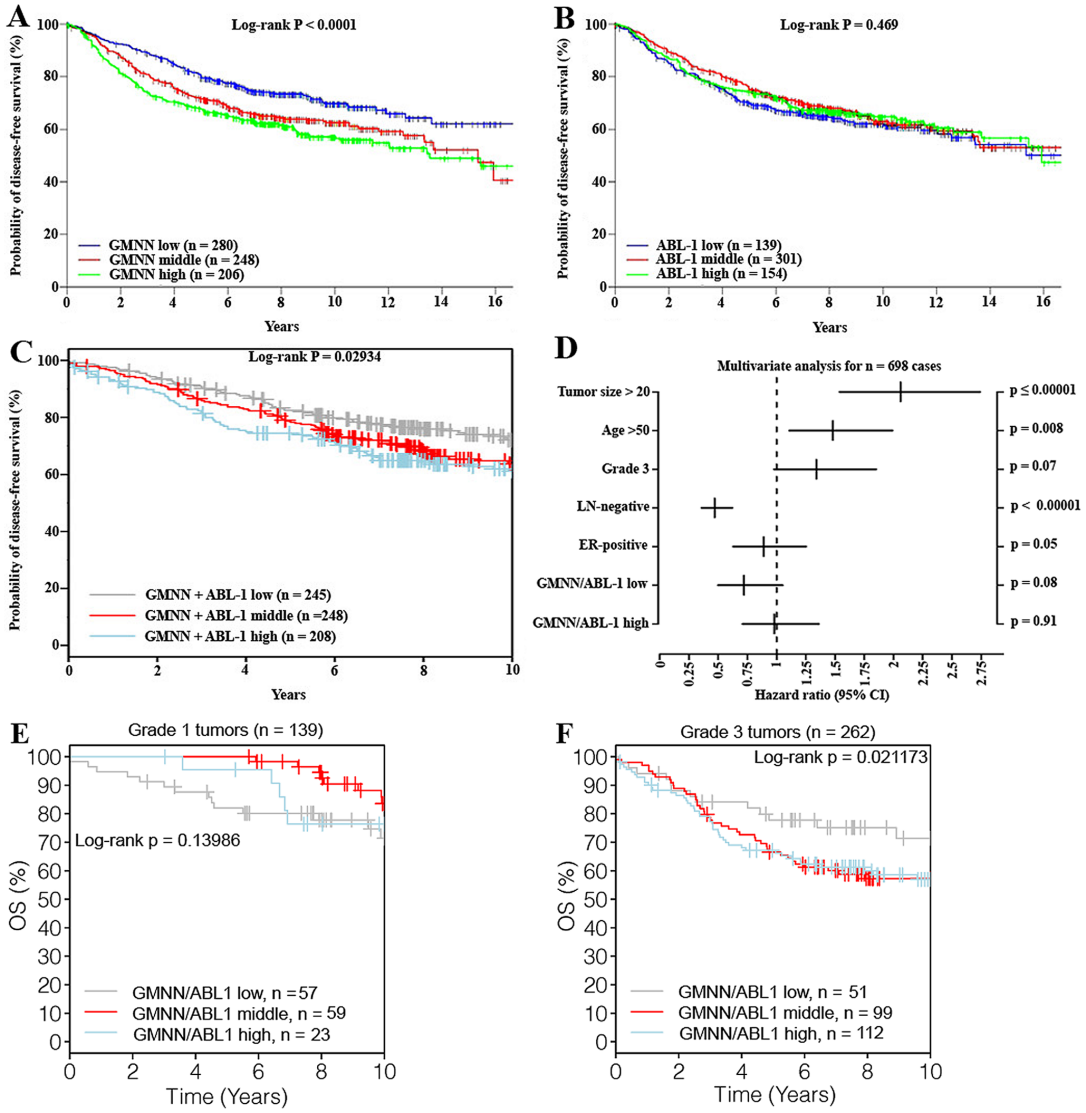
Geminin/c-Abl overexpression decreases the overall survival and increase hazard ratio in breast cancer patients. (A) Kaplan-Meier analysis for disease free survival (DFS) of patients expressing high, middle or low levels of geminin in their breast tumors. (B) Kaplan-Meier analysis for DFS of patients expressing high, middle or low levels of c-Abl in their breast tumors. (C) Kaplan-Meier analysis for DFS of patients expressing high vs. middle vs. low levels of geminin + c-Abl in their breast tumors. (D) Multi-variants analysis of the hazard ratio (estimated mean survival with 95% CI) plotted against tumor size, age, grade 3, lymph-node (LN)-negativity, ER-positivity. Kaplan-Meier analysis for overall survival (OS) of patients expressing high, middle or low levels of geminin + c-Abl in their grade 1 (E) or grade 3 (F) breast tumors.

Moreover, using the GOBO bioinformatics resource (for size and inclusion of samples, see Methods) we attempted to measure the association of geminin/c-Abl co-overexpression with disease-free survival (DFS), overall survival (OS) and distant metastasis-free survival (DMFS). Significant decrease in DFS was measured in tumors expressing high (n = 206) and moderate (n = 248) compared to low (n =  280) levels of geminin (*p*<*0.0001*, Fig. 6A). No such decrease was measured when tumors expressing high (n = 129), moderate (n = 301) and low (n = 154) levels of c-Abl were compared (*p* = *0.469*, Fig. 6B). However, significant decrease in DFS was detected when high (n =  208) and moderate (n = 248) geminin + c-Abl expressing tumors were compared to low (n = 245) geminin + c-Abl expressing tumors (*p* = *0.029034*, Fig. 6C).

Meta-analysis performed on a cohort of n = 698 for overall survival hazard ratio (95% CI) showed significant decrease in OS with tumor size (*p*≤*0.00001*, Fig. 6D), age (*p* = *0.008*, Fig. 6D), LN-negativity (*p*<*0.00001* vs. LN-positivity *p* = *0.4374*, Fig. 6D), ER-positivity (*p* = *0.00521* vs. ER-negativity *p* = *0.66236*, Fig. 6D). Finally a strong correlation between high geminin/c-Abl co-expression and lower OS was observed in G3 (*p* = *0.021173*, n = 262, Fig. 6F), and not G1 (*p* = *0.13986*, n = 139, Fig. 6E) tumors. When DMFS as an endpoint with 10 year censoring for the combined geminin/c-Abl overexpression was studied, worse clinical outcomes was observed for HER2^+^ or normal-like tumors (*p*<*0.05*, data not shown). Taken together, these data show that geminin/c-Abl-positivity correlates with adverse breast tumor status and outcomes, and thus could be used as a novel diagnostic biomarker for aggressive breast tumors.

### Geminin overexpressing tumors are sensitive to decreasing c-Abl expression or activity

We showed earlier that geminin overexpressing cells (using induced Gem9 cells) develop aggressive, invasive and aneuploid mammary xenograft/orthotopic tumors in SCID mice [10]. To study whether these tumors are sensitive to decreasing c-Abl expression and/or activity, we injected 6-8 weeks old SCID mice in mammary fat pads with Gem9 cell clones expressing inducible control (n = 20), geminin (sh-geminin, n = 10) or c-Abl (sh-c-Abl, n = 10) shRNAs. Ten mice injected with Gem9 expressing control shRNA were maintained on regular water, while the other 10 mice and the mice injected with Gem9 expressing sh-geminin and sh-c-Abl were maintained on Dox-containing drinking water. Tumor development was monitored weekly by Xenogen *in vivo* imaging, and once formed their size was measured daily with caliper.

**Figure 7:**
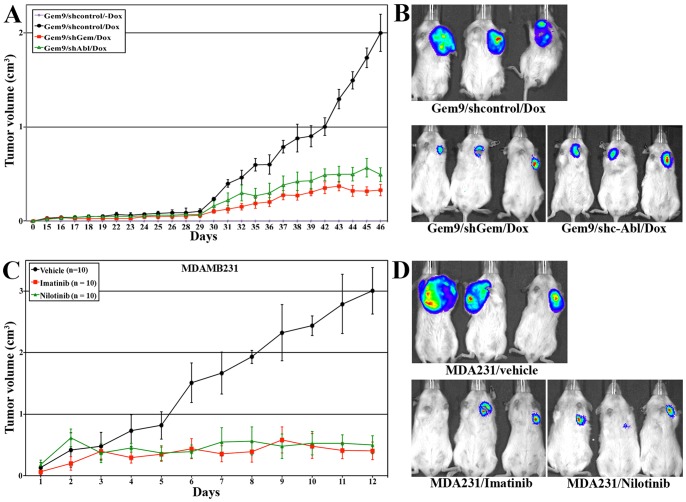
The effect of c-Abl silencing or inactivation on geminin-driven mammary tumors. (A, left) The volumes of geminin-driven orthotopic tumors following expression of sh-control (black line, n  = 10), shc-Abl (green line, n = 10) or sh-geminin (red line, n = 10) in Gem9 cells. All mice were kept on doxycycline containing drinking water throughout the experiment. A set of 10 mice were injected with Gem9 cells and kept on no doxycycline water (purple line). (A, right) Representative luciferase images taken at day 45 of tumors shown in (A, left). (B, left) The volumes of MDAMB231 orthotopic tumors following treatment with vehicle (black line, n  = 10), imatinib (red line, n = 10) or nilotinib (green line, n = 10). (B, right) Representative luciferase images taken at day 12 of tumors shown in (B, left).

No tumors were detected in mice maintained on Dox-free water (see purple line in Fig. 7A), whereas palpable tumors began to develop in all mice on Dox ∼30 days later. Tumors developed in mice injected with Gem9 + control shRNA grew exponentially and reached ∼2 cm^3^ by day 45 (see black line in Fig. 7A, and 7B upper panel). In contrast, mice injected with Gem9 + sh-geminin (red line in Fig. 7A and 7B lower panel, left) or Gem9 + sh-c-Abl (green line in Fig. 7A and 7B lower panel, right) cells, as expected, remained at 0.25-0.5 cm^3^ until the end of the experiment at day 45.

We recently also showed that geminin overexpression is required for mammary tumors maintenance using the aggressive TNBC breast cancer cell line, MDA-MB-231 [10]. To evaluate whether c-Abl activity is also required for maintenance of these tumors, 30 SCID mice were injected in mammary fat pads with MDA-MB-231 cells. Starting on day 1 post-injection mice were treated with vehicle (n = 10), 50 mg/kg imatinib (n = 10) or 4 mg/kg nilotinib (n = 10) daily (weekends off). Tumors reached ∼3 cm^3^ in 2 weeks in vehicle treated mice (black line in Fig. 7C, and 7D, upper panel), but remained at ∼0.5 cm^3^ in imatinib and nilotinib treated mice by the same time (red and green lines in Fig. 7C, respectively and Fig. 7D, lower panels). Another 30 mice were injected in the fat pads with Gem9 cells, and maintained on Dox-containing water until palpable tumors were observed (∼day 30), at which time received vehicle (n = 10), 50 mg/kg imatinib (n = 10) or 4 mg/kg nilotinib (n = 10) daily (weekends off). Tumors grew to ∼2 cm^3^ by day 50 in vehicle treated mice (Fig. S4A and data not shown), but only to ∼0.25 cm^3^ by day 50 in imatinib (Fig. S4B and data not shown) or nilotinib (Fig. S4C and data not shown) treated mice.

### Suppressing c-Abl expression or activity diminishes geminin expression in orthotopic mammary tumors

**Figure 8:**
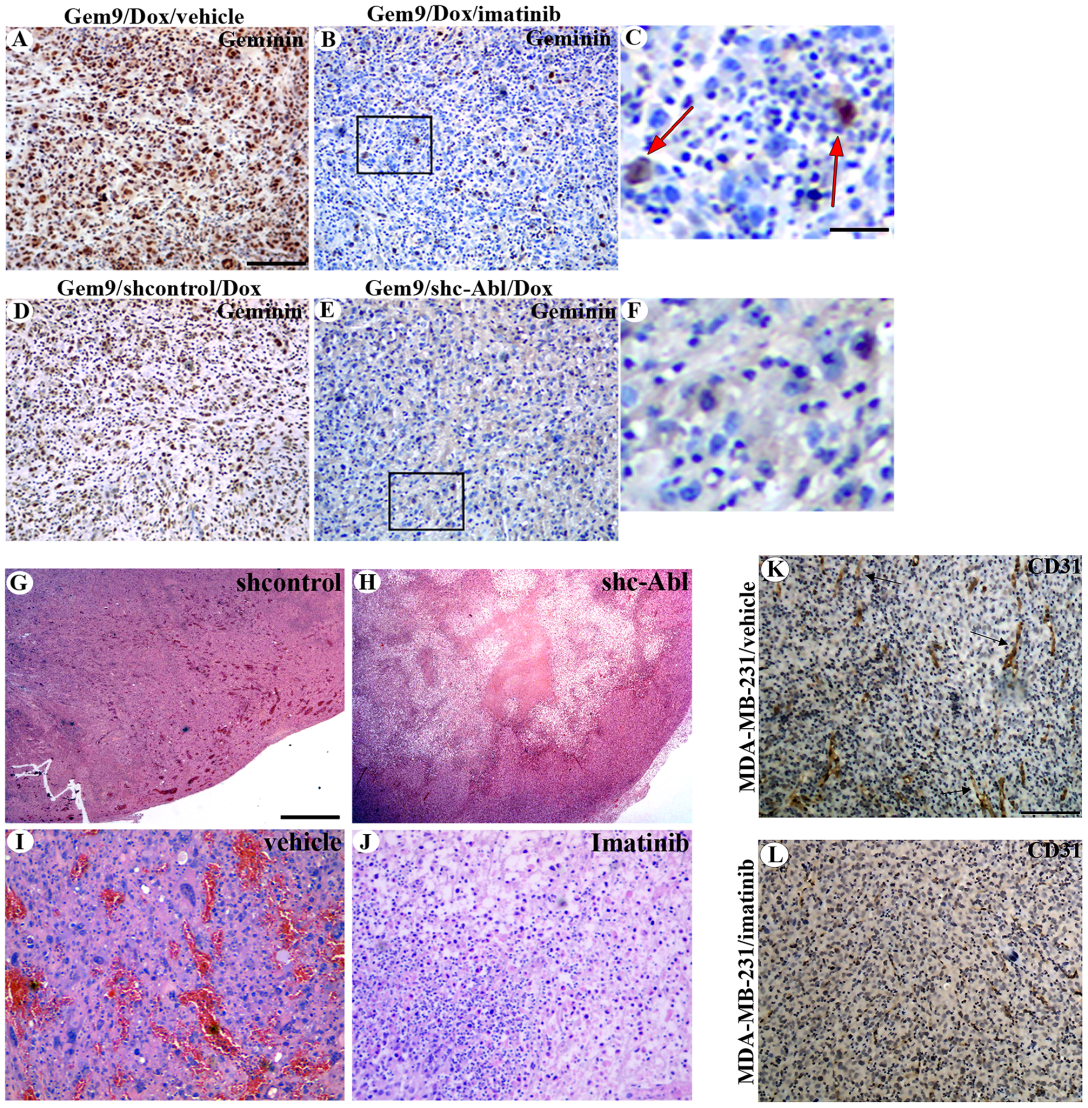
Histological and immunohistochemical analysis of geminin overexpressing mammary tumors following c-Abl silencing or inactivation. Immunohistochemical staining of induced Gem9 tumors treated with vehicle (A), imatinib (B and C), expressing control shRNA (D) or c-Abl shRNA (E and F). H&E stained induced Gem9 tumor expressing control (G) or c-Abl (H) shRNA. H&E stained induced Gem9 tumor following treatment with vehicle (I) or imatinib (J). Scale bar in A, B, D, E, I and J = 200 µm, in C and F = 50 µm and in G and H = 500 µm. (K and L) CD31 staining on section from MDA-MB-231 tumors treated with vehicle (arrows, K) or imatinib (E) treated geminin-driven tumors. Scale bar = 200 µm.

To study whether the regression in orthotopic geminin-overexpressing tumors following reduced c-Abl expression or activity, could be due to diminished geminin expression, these tumors were stained with anti-geminin antibody. Unlike controls that expressed geminin in every cell (Fig. 8A and 8D), imatinib treated (Fig. 8B, 8C) or c-Abl shRNA expressing tumors (Fig. 8E and 8F) showed almost complete absence of geminin-positive cells. Similar results were obtained with tumors developed in mice treated with nilotinib instead (not shown).

Furthermore, this was correlated with lack of cellularity and neo-vasculature detected in the H&E stained sections of sh-c-Abl expressing (Fig. 8H) or imatinib treated tumors (Fig. 8J) compared to controls treated tumors (Fig. 8G and 8I). In fact, high percentage of angiogenesis could be seen in tumors developed using MDA-MB-231 treated with vehicle (Fig. 8K) compared to those treated with imatinib (Fig. 8L) as detected using IHC for mouse specific endothelial marker; CD31. Taken together, these data clearly show that like *in vitro*, *in vivo* c-Abl depletion or inactivation reduces geminin protein stability, which leads to death of tumor cells that overexpress geminin and to tumor regression. These data also highlight the fact that c-Abl inactivation could be pursued to treat aggressive breast cancer stratified as overexpressing geminin/nuclear c-Abl.

### Geminin-driven orthotopic tumors also express nuclear c-Abl

**Figure 9:**
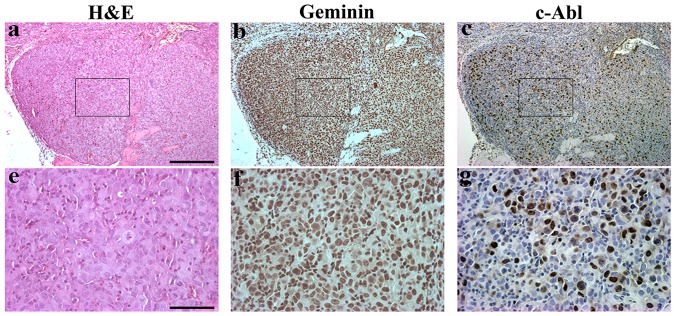
Histological and immunohistochemical analysis of geminin overexpressing mammary tumors. (a and e) Representative H&E stained sections from induced Gem9 orthotopic mammary tumors. (b and C) and (f and g) adjacent sections to those shown in (a and e) stained with geminin (b and f) or c-Abl (c and g). Scale bars in a-c = 500 µm and d-f =  100 µm.

To study the localization of c-Abl in geminin-driven tumors, we IHC stained orthotopic geminin-driven tumors (Fig. 9a and c) with anti-geminin and anti-c-Abl antibodies. In keeping with human primary tumors (see Fig. 5), tumors developed using geminin-overexpressing cells (Fig. 9b and f) expressed almost exclusively nuclear c-Abl (Fig. 8c and g). These data perhaps suggest that *in vivo*, during the evolution of geminin-overexpressing breast tumors, a pressure is exerted that makes them express only nuclear c-Abl (see discussion).

### Imatinib as a treatment option for geminin overexpressing tumors

**Figure 10:**
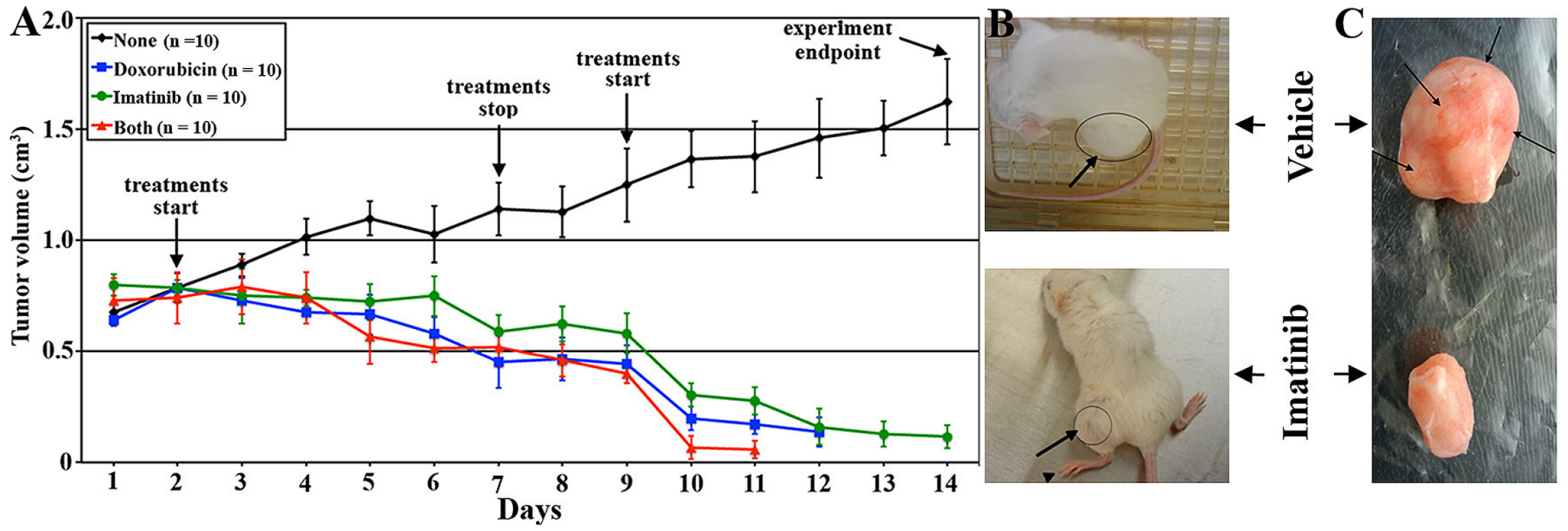
The efficacy of imatinib against geminin overexpressing tumors. (A) Tumors developed orthotopically or subcutaneously using induced Gem9 cells were treated when reached 0.5–0.75 cm^3^ with doxorubicin (blue line, n = 10), imatinib (green line, n = 10) or both (red line, n = 10) daily (weekend off) for 14 days. Black line shows vehicle treated tumors (n = 10) as described above. Representative subcutaneously (B) or orthotopically (C) developed tumors following vehicle (uppers) or imatinib (lower) treatments.

Finally, to evaluate the utility of imatinib as a treatment option for geminin-overexpressing tumors, 40 SCID mice were injected in mammary fat pads and 40 SCID mice were injected subcutaneously with Gem9 cells. All 80 mice were maintained on Dox-containing water until tumors reached 0.5-0.7 cm^3^ by ∼day 35. At that time mice in both sets were divided into 4 groups that received vehicle (n = 10), doxorubicin (5 mg/kg/day, n = 10), imatinib (50 mg/kg/day, n = 10) or both (n = 10). In both sets, tumors treated with vehicle grew exponentially to reach >1.5 cm^3^ 2 weeks later (Fig. 10A, and upper in 10B and 10C). Tumors treated with doxorubicin (Fig. 10A) or imatinib (Fig. 10A and lower in 10B and 10C) significantly regressed. Interestingly, at these clinically relevant concentrations, imatinib was much less toxic than doxorubicin (Fig. 10A). Overall, our data support the notion that anti-c-Abl therapy, such as imatinib/nilotinib could be used to treat geminin-overexpressing tumors, which express nuclear c-Abl.

## Discussion

Cytokinesis failure leads to aneuploidy and cause cancer progression [41], [42]. Recently, we showed that geminin overexpression induces cytokinesis skipping and aneuploidy, *in vitro*, and formation of aggressive tumors, *in vivo* [10]. We discovered that this was, at least in part, due to geminin overexpression ability to prematurely inactive two mitotic enzymes, namely, TopoIIα and AurB kinase [41]–[43]. Overexpression of WT geminin induced aneuploidy, while overexpression of any single Y-to-A mutant geminin promoted apoptosis instead of aneuploidy [10], supporting the notion that only WT geminin Y-phosphorylated (simultaneously on all three tyrosine residues Y98, 111 and 150) has tumor inducing abilities. This also implicates geminin upstream kinase(s) in geminin-overexpression ability to induce tumorigenesis. It is possible that cells become addicted to oncogenic effects exerted by the overexpressed tyrosine phosphorylated WT-geminin (on all Ys), and when c-Abl (or the other kinases) is silenced or inhibited these cells die [10]. Indeed, only cells overexpressing WT geminin showed increased Bcl-2 and Bcl-xL expression [10].

One of the interesting observations of this study is the fact that only c-Abl isolated from G_2_/M cells could phosphorylate geminin, *in vitro*. It is possible that unlike S-phase c-Abl, G_2_/M c-Abl itself is differentially modified, that c-Abl is bound to another kinase in this phase that phosphorylates geminin, or that in the two phases c-Abl reside in two different compartments; cytoplasmic (in S-phase) vs. nuclear (in G_2_/M) and in the cytoplasmic fraction c-Abl is inhibited. Although we are in favor of the first, experiments to sort out between all three possibilities are currently undergoing in the laboratory.

Another interesting observation is how c-Abl is retained in the nucleus in aggressive breast cancers *in vivo*. A clue might be in the analysis of the orthotopic geminin-overexpressing tumors we generated. The fact that in these tumors c-Abl was also predominantly nuclear suggests that *in vivo* geminin overexpression precedes c-Abl nuclear retention. c-Abl phosphorylation on T735 by the monopolar spindle 1 kinase (Mps1/TTK) [44] induces its binding to 14-3-3 (most likely 14-3-3σ) and retention in the cytoplasm [45]. Upon mitogenic or geno-/cell-toxic stimulation of cells, 14-3-3σ is phosphorylated by JNK, which leads to the release of c-Abl and its nuclear translocation [44]. We propose that in geminin-overexpressing tumors, T735 in c-Abl is mutated, that Mps1/TTK kinase is activated, that JNK is over-activated or that 14-3-3σ expression is decreased. Only evidence for the latter has already been shown [46]. Nevertheless, we currently investigate all these possibilities using geminin overexpressing TNBC patients’ samples.

Another aspect of our observations that requires commenting is the fact that in most situations c-Abl nuclear translocation was associated with enhanced apoptosis [22], [23] and not tumorigenicity. In our hands, we saw an upregulation of c-Abl in ∼90% of all breast tumor samples analyzed and in ∼50% of those the expression was exclusively nuclear. It is possible that the translocation of c-Abl from the cytoplasm to the nucleus is involved in apoptosis induction in some circumstances [22], [23], while in others in induction of cell survival, proliferation and transformation. For instance, c-Abl translocation from the cytoplasm to the nucleus in response to serum and phosphorylation of DGKα promotes the latter cytoplasmic translocation and induction of cell proliferation and transformation [47]. C-Abl nuclear translocation was associated recently with phosphorylation of PCNA and induction of cell proliferation [48], AIB1 and tumor formation [49], and c-Jun or c-Fos and promotion of cell proliferation [50], [51]. It is possible that retention of c-Abl in the nucleus and/or expression of exclusively nuclear c-Abl protein can serve as an oncogene [52] as we showed here. Indeed, others have also shown decreased OS in c-Abl overexpressing breast tumors cohorts [53].

Finally, several clinical trials showed no efficacy for imatinib as a mono-therapy in breast cancer patients. Especially disturbing is a study showing imatinib to increase tumor size in athymic mice injected with MA-11 cells [54]. However, MA-11 cells were also resistant to treatment with cisplatin and doxorubicin [55], which might suggest that this is an inherent trait of these cells [55], [56]. Imatinib *plus* paclitaxel trail actually showed benefit in a phase I clinical trial in patients with advanced or metastatic solid tumors refractory to standard therapy [57], and imatinib *plus* capecitabine also showed benefit in a phase II trial of unselected breast cancer patients [58]. It is possible that lack of obvious efficacy in some unselected trials with imatinib reflects just that “the un-selection”. We propose that stratifying patients according to their tumor expression of geminin and nuclear c-Abl could increase the efficacy of imatinib. Even in these stratified cohorts, which according to our calculation should reflect ∼50% of all patients, only a sub-group of those will be the most responsive to imatinib, most likely due to other confounding factors we have yet to identify.

Collectively, our data define a novel role for geminin as a breast cancer oncogene that promotes breast cancer development and progression, and implicates nuclear c-Abl as an activator of this function. Many promising targeted therapies for solid tumors have failed to show efficacy, because they do not reach their targeted population due to lack of biomarkers stratification of these patients [59]. TNBC affects minorities disproportionately, and is over-represented in the African-American population in USA. Our discovery that geminin/nuclear c-Abl are frequently overexpressed in TNBCs, and that geminin-overexpressing tumors are highly sensitive to imatinib in pre-clinical mouse model, justify the use of this combination (geminin/nuclear c-Abl) as a biomarker to stratify TNBC patients who may respond to a treatment regimen including imatinib; a drug not commonly used in breast cancer.

## Methods

### Cell Culture and drug treatment

Breast cancer cell lines were maintained in RPMI medium (Invitrogen) supplemented with 10% FBS and antibiotics. HME cells maintenance described earlier [60]. Doxycycline, MG132, colcemid, cycloheximide and TBB were from Sigma, all other drugs were from Toronto Research Chemicals Inc.

### Antibodies

A mouse anti-geminin monoclonal antibody [8] and rabbit polyclonal anti-pY150-geminin were developed in our laboratory, 2 different mouse monoclonal anti-c-Abl were used and gave essentially identical results, one was from (Cell Signaling, #2862) and the other from (Santa Cruz, sc-23), rabbit anti-Cdt1 (abcam, ab22716), rabbit anti-Sp1 (Santa Cruz, sc-14027), rabbit anti-p-H3^S10^ (Upstate, 06-570), mouse anti-Actin (Calbiochem, cat. # cp01), mouse anti-p-CrkII [Y221, Cell Signaling, #3491] [61], rabbit anti-H2B (abcam, ab18977), rat anti-CD31 (abcam, ab7388), and goat anti-Arg (Santa Cruz, sc-6356).

### HME cells synchronization protocol

Removing growth factors from the culture medium for 72 h synchronizes HME cells in G_0_/G_1_. Re-addition of growth factors to the cultures for 16, 22 and 26 h synchronizes cells in S-, G_2_/M- and M/G_1_, respectively (see Fig. S1). PI or FITC-conjugated anti-BrdU FACS analysis was performed as in [60].

### Chromatin, nuclear and cytoplasmic extracts purification and immunoprecipitation

Protocol for chromatin extraction was described earlier in [60]. To isolate nuclear vs. cytoplasmic from the same cells, cells were washed with ice cold PBS, re-suspend in buffer 1 (containing: 10 mM HEPES, 10 mM KCl, 0.5 mM DTT, 1% NP-40) and incubated 10 min at 4°C with gentle agitation, followed by centrifuge for 2 min at max speed and the supernatant was saved as cytoplasmic extract. Nuclear pellet was re-suspended in buffer 2 (containing: 20 mM HEPES, 20% Glycerol, 500 mM KCl, 0.2 mM EPTA, 0.5 mM PMSF, 0.5 mM DTT, 1.5 mM MgCl2); incubated 15 min at 4°C with gentle agitation, sonicated and then spun down at max speed to remove membrane faction and the supernatant was saved as nuclear extract. In all immunoblotting experiments, equal concentration (usually 25 µg) from each extract is loaded on the gel.

### Constructs, transit and stable transfection

Twenty µg of pcDNA3.1 (control) or pcDNA3.1-K290R-c-Abl and pcDNA3.1-P242/249A-c-Abl [26] were transfected in 50% confluent HME cells using Lipofectamine PLUS reagent (Invitrogen) in 10 cm^2^ dishes. Clontech kit Rev-Tre/Tet-ON inducible system was used. Wild type geminin cDNA was amplified from HME total RNA using primers that amplify the whole cDNA including portions from the 5′- and the 3′-UTRs, mutagenesis was done using QuickChange Site-Directed Mutagenesis Kit (Stratagene) and primers. GST-wild type, Y98A, Y111A, Y150A expression plasmids were also generated using PCR technique in the pGEX-4T2.

### RNA interference experiment

Geminin siRNA was described in [8]. A pre-synthesized c-Abl siRNA from (Dharmacon) was used. Transfection of siRNAs in breast cancer as well as HME cells was performed using Oligofectamine 2000 (Invitrogen) according to the manufacturer’s instructions. Shcontrol/ and shc-Abl/GFP was a kind gift from Dr. Lindsey D. Mayo (Wells Center for Pediatric Research, Indianapolis).

### Virus and protein expression

Retroviruses production was done using standard protocols. After infection 10 hygromycin selected clones were tested for the expression of the exogenous geminin using anti-His Western blot. Clone #9 and 10 (Gem9 and Gem10) were chosen for the analysis described here. The GST-fused geminin was expressed in competent bacteria “One shot BL-21 star (DE3)pLysS” (Invitrogen), induced with IPTG and purified on Glutathione SepharoseTM 4B beads (GSSH), and eluted from the beads using 10 mM of Glutathione in 50 mM Tris–HCl pH 8.0.

### Real time RT/PCR assays

Total RNA was isolated after treatments using TRIzol reagent (Invitrogen) and treated with a DNA-free kit (Ambion, Austin, TX) to eliminate genomic DNA contamination. Quantitative RT/PCR analyses were performed according to standard protocols using iQ Sybergreen Supermix using the primers; *geminin*: forward 5′- CGGGATCCATGAATCCCAGTATGAAGCAGAAACAAGAA-3′ and reverse 5′- ACGCGTCGACTCATATACATGGCTTTGCATCCGTA, *c-Abl*: forward 5′-GATACGAAGGGAGGGTGTACCA-3′, reverse 5′-CTCGGCCAGGGTGTTGAA-3′, *GAPDH:* forward 5′-GGACCTGACCTGCCGTCTAG-3′ and reverse 5′-TGGTGCTCAGTGTAGCCCAG-3′. GAPDH are common sequences adopted from public literature. Triplicate C_T_ values were analyzed in Microsoft Excel using the comparative C_T_ (ΔΔC_T_) method as described by the manufacturer (Applied Biosystems). The amount of target (2^-ΔΔCT^) was obtained by normalization to an endogenous reference (GAPDH RNA) and relative to a calibrator.

### Metaphase spread

A 100 ng/ml colcemid was added directly to cultures and cultures were incubated for 1 hrs then cells were trypsinized cells and washed. Cells were then re-suspended in the residual PBS and 0.075 M KCl was added to cells drop wise up to 10 ml, and cells were then incubate in a water bath at 37°C for 5-10 mins. Cells were then centrifuge at 900rpm for 5 minutes and KCl was removed and cells were gently resuspended in the residual KCl. To cells a 5 ml of freshly prepared fixative medium (3∶1 Methanol/Acetic acid) was added drop wise and carefully mix. Cells were centrifuge at 900rpm for 5 minutes and the fixative medium was removed, and this step was repeated two extra times. Finally, the fixative medium was removed and a new 300 µl of the fixative was added. The cell suspension was drop drops from ∼18 inches onto angled, humidified microscope slide. Slides were then air-dry at least 10 mins in room temperature, then PI stained.

### TUNEL apoptosis detection assay and soft agar colony formation assay

Detection of apoptotic cells was done using “In situ Direct DNA Fragmentation (TUNEL) Assay Kit (ab66108)” according to manufacture procedures. A 1% Noble Agar (Difco) and 2 X DMEM/F12 with additives were prepared and then cooled to 40°C in a water bath for 30 minutes. Equal volumes of the two solutions were mixed to give 0.5% Agar in 1 X DMEM/F12 with additives. This solution (1.5 ml) was added to wells in 6 well plates and allowed to settle. A 0.7% Agar was prepared and 2 X DMEM/F12 + additives were brought to 40°C in a water bath. A 5,000 cells/well in 3 ml of the above solution was added to each well and incubated at 37°C in humidified incubator for 2-3 weeks in the presence or absence of 2 µg/ml Dox and 10 µM imatinib. Colonies formed were then stained with 0.5 ml of 0.005% Crystal Violet for 1 hour, counted using a dissecting microscope. HEK293T cells were used as positive control and IMR90 cells as negative control.

### Tissue samples and immunohistochemical (IHC) analysis of paraffin-embedded tumor samples

A University of Hawaii IRB committee approved the use of human tumor sample. A training cohort was a commercial TMA (Biomax.us, n = 511 samples) containing normal/cancer adjacent tissues (n = 66), ductal carcinoma *in situ* (DCIS, n = 180), invasive (n = 100), and metastatic (n = 165) breast tumor samples and a confirmation cohort, consisted of disease-free adult tissues (including; kidney, liver, placenta, spleen and mammary tissues) and a conformational cohort (n = 326, breast tumor samples, different stages) acquired from the Hawaiian *Surveillance, Epidemiology and End Results* (SEER) collection constructed in quadruplicate, each containing one sample from a different region of a tumor at 4 µm were used.

### IHC staining soring

Immunostained slides were scored using a modified protocol of the one described previously [62], [63]. In brief, stained sections were evaluated by light microscopy. Positivity score was assigned by counting positive cells in at least 10 high power fields of each tumor section and the scored was estimated as follow: 0  =  no staining (<1% of the cells stained); 1  =  weak (1–10% of the cells stained); 2  =  medium (10–50% of the cells stained); 3  =  strong (>50% of the cells stained). Next, an intensity score was assigned, in which the average intensity of positive tumor cells is represented as 0 = none, 1  =  weak, 2  =  intermediate, and 3  =  strong. The positivity and intensity scores were then added to obtain a total score, which ranged from 0 to 6. A pathologist scored slides blindly.

### RNA and RT/PCR on breast tumor samples

This study also utilizes RNA from previously published cohort of primary sporadic invasive breast carcinomas representative of breast cancer in the general population [64]–[66]. This included 39 specimens of breast tissues were used, of those 5 normal organoids, 7 luminal A, 9 luminal B, 11 Her2^+^ and 7 TN/BL tumors. Stage, grade, tumor size, and ER, PR, and HER-2 expression were determined as described [65]. Also from xenograft tumors 4 µm sections were prepared that was processed for antigen retrieval technique was carried out by microwave treatment of the slides in sodium citrate buffer (pH 6.0) for 20 min.

### OS and DMFS analysis

The association was investigated for stratified patient cohorts using overall survival (OS) and distant metastasis-free survival (DMFS) in 7 major subgroups: all tumors, ER-positive, ER-negative, LN-psoitive, LN-negative, untreated patients and patients systematically treated with tamoxifen. In brief, the GSA-tumor analysis application module (GSA-Tumor) in GOBO was used to generate Kaplan-Meier survival analysis and was based on a 1881-sample breast tumor set comprised 11 public data sets analyzed using Affymetrix U133A arrays. In this analysis, if a gene set consists of multiple gene probes an average expression is computed for the total gene set, taking consideration to gene weights if supplied. In addition, the Kaplan-Meier survival analysis was also determined for the gene set in 21 subgroups for 1379 cases with DMFS follow-up.

### *In vivo* tumorigenicity assay

The University of Hawaii or the University of Mississippi Medical Center IACUC committees approved all animal experiments. Six- to eight-week-old anaesthetized immune-compromised athymic SCID (NOD.CB17-*Prkdc* ^scid^/J, Jackson Laboratory) mice were injected with 5×10^6^ cells resuspended in 200 µl of HME medium/matrigel (1∶1) using a 25-gauge needle either subcutaneously in the left thigh or orthotopically in the 2^nd^ mammary gland. Tumor initiation was defined as the time when tumors were 3 mm in diameter. Mice were sacrificed when the tumors grew to ∼1.5 cm in diameter or after 12wk of monitoring. Tumor volume was calculated with the formula 4/3πr^3^ (where r is the tumor radius). At the end of the experiments, mice were sacrificed by compressed 100% CO_2_ gas. Mice were placed in chamber without pre-charging and gas was introduced from a cylinder source that allows the inflow of gas to the induction chamber to be controlled. A fill rate of about 10% to 30% of the chamber volume per minute with gas, added to the existing air in the chamber achieved rapid unconsciousness with minimal distress to the animals. Tumors were then dissected out, weighed and then fixed in formalin, cut at 4 µm for histological and immunohistochemical analysis.

### *In vivo* measurement and imaging of orthotopic or subcutaneous tumors

Tumor formation was analyzed with IVIS luciferase machine (Xenogen) weekly and tumor size was measured every 3^rd^ day by caliper (Life Sciences instruments). To analyze tumor formation using the *in vivo* system, mice were i.p. injected using 30G needle with 100 µl of D-luciferin solution (Xenogen) prepared at 15 mg/mL in PBS. Mice were then anesthetized using a mix of oxygen and isoflurane gas. Anesthetized animals were maintained sleep during the imaging procedures by placing the animal right side (injection side) up and its nose in a nose cone with a flow of anesthesia gas and take a picture of the tumors.

### *In vivo* drug treatments

Geminin overexpressing cells were injected subcutaneously or orthotopically (in mammary gland) in SCID mice as described above. Mice with tumors at different intervals/sizes were treated using 5 mg/kg/day doxorubicin, 50 mg/kg/day imatinib, 4 mg/kg/day nilotinib daily (weekend off) by gavage injection. Tumor size was measured daily using caliper. At the end of the experiments tumors were dissected out, weighed and fixed in formalin, cut at 4 µm for histological and immunohistochemical analysis.

### Overall survival, distant metastasis free survival and hazard ration analysis

Data source for disease-free survival used was the GOBO bioinformatics resource, the association of the combined set with outcome was investigated for stratified patient cohorts using overall survival (OS) and distant metastasis-free survival (DMFS) in five major subgroups: all tumors, ER-positive tumors, ER-negative tumors, systematically untreated patients and patients treated with tamoxifen. The GSA-Tumor Analysis application module (GSA-Tumor) in GOBO is based on a 1881-sample breast tumor set comprised 11 public data sets analyzed using Affymetrix U133A arrays. In the GSA-Tumor, if a gene set consists of multiple genes an average expression is computed for the total gene set, taking consideration to gene weights if supplied. Furthermore, the Kaplan-Meier survival analysis was determined for the gene set in 21 subgroups for a total of 1476 cases with DMFS or OS follow-up from the GEO study as fellows: GSE1456, sample size: 159, GSE2034, sample size 286, GSE2603, sample size 121, GSE4922, sample size 249, GSE6532, sample size 327, GSE7390, sample size 198, GSE12093, sample size 136.

### Statistical analysis

Comparisons of treatment outcomes were tested for statistical differences using the Student t-test for paired data. The association of mRNA transcript expression with various clinico-pathologic parameters was also analyzed. Statistical significance was assumed at a P-value are * ≤ 0.05, ** ≤ 0.01 and *** ≤ 0.001.

## Supporting Information

Figure S1 Cell cycle analysis of HME cells synchronized in different phases, isolation of geminin S and G_2_/M phase protein complexes and effects of c-Abl siRNA and shRNA. FACS analysis of HME cells grown in no growth factors containing medium for 72 h (G_0_/G_1_, A), after growth factors addition for 16 h (S phase, B), 22 h (G_2_/M phase, C) and in 26 h (M/G_1_, D). (E) S and G_2_/M phase HME cells extracts were IPd with geminin antibody, run on a gel, proteins cut from the gel and subsequently micro-sequenced. * Shows the position of geminin. (F) The expression levels of c-Abl or Arg in MDA-MB-231 cells transfected with si-c-Abl (left), or in inducible Gem9 cells stably expressing sh-c-Abl (right).
(TIF)

Figure S2 The effect of several c-Abl inhibitors on c-Abl expression and activity, and geminin Y150 phosphorylation by c-Abl, *in vivo* induces geminin stability. (A) The expression levels c-Abl, p-CrkII and geminin in inducible Gem9 cells treated with 0, 10 and 50 µM of imatinib (left), nilotinib (middle) or dasatinib (right) for 24 h. (B) The expression of Myc-3Y-to-E- and not Myc-WT-geminin or Myc-3Y-to-A-geminin in Hs578T. (C) The re-expression of geminin protein in Hs578T cells reconstituted with WT or CA c-Abl detected using an anti-total geminin or anti-p-Y150 antibodies. Note that imatinib treatment significantly decreased the levels of total and to even higher extent the levels of p-Y150 geminin protein in these reconstituted cells (see Tables below). (D) Expression of total or p-Y150 geminin in MDA-MB-231 cells transfected with si-control or sic-Abl. The level of reduction in total or p-Y150-geminin is presented in Table below the figure. (E) Quantification of the cycloheximide effect on endogenous and exogenous geminin shown in Fig. 4H.
(TIF)

Figure S3 Imatinib kills geminin-overexpressing cells. Representative FACS analysis of MDA-MB-231 cells treated with vehicle (or expressing si-control, A), expressing si-c-Abl (B) or treated with imatinib (C). Note the increased sub-G1 fraction in c-Abl silenced or inactivated cells.
(TIF)

Figure S4 The effect of c-Abl inactivation on geminin overexpressing tumors. Representative images of inducible Gem9 tumors developed in SCID mice following treatment with vehicle (A), imatinib (B) or nilotinib (C).
(TIF)

Table S1 The association between geminin overexpression and overexpression of nuclear or cytoplasmic c-Abl in breast tumors.
(DOCX)

Table S2 Relationships between geminin expression and tumor characteristics in Her2^+^ and TN/BL breast cancer tumor samples.
(DOCX)
