# Supplementary figures and images for: Geminin Overexpression Promotes Imatinib Sensitive Breast Cancer: A Novel Treatment Approach for Aggressive Breast Cancers, Including a Subset of Triple Negative
Source: PLoS One. 2014 Apr 30;9(4):e95663. doi: 10.1371/journal.pone.0095663 (PMC4005756; doi:10.1371/journal.pone.0095663)

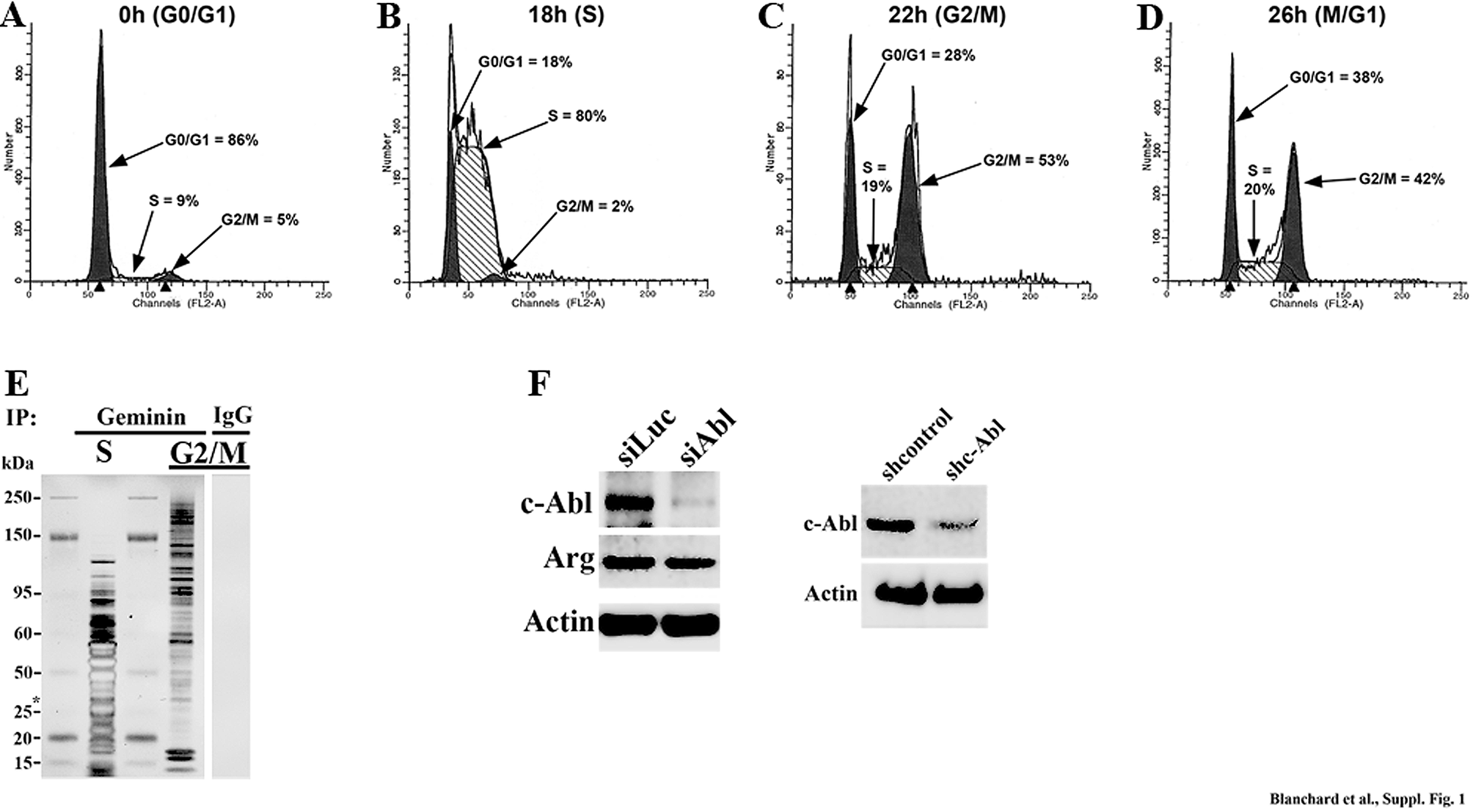

Supplement: Figure S1 — Cell cycle analysis of HME cells synchronized in different phases, isolation of geminin S and G2/M phase protein complexes and effects of c-Abl siRNA and shRNA. FACS analysis of HME cells grown in no growth factors containing medium for 72 h (G0/G1, A), after growth factors addition for 16 h (S phase, B), 22 h (G2/M phase, C) and in 26 h (M/G1, D). (E) S and G2/M phase HME cells extracts were IPd with geminin antibody, run on a gel, proteins cut from the gel and subsequently micro-sequenced. * Shows the position of geminin. (F) The expression levels of c-Abl or Arg in MDA-MB-231 cells transfected with si-c-Abl (left), or in inducible Gem9 cells stably expressing sh-c-Abl (right). (TIF) [file pone.0095663.s001.tif]

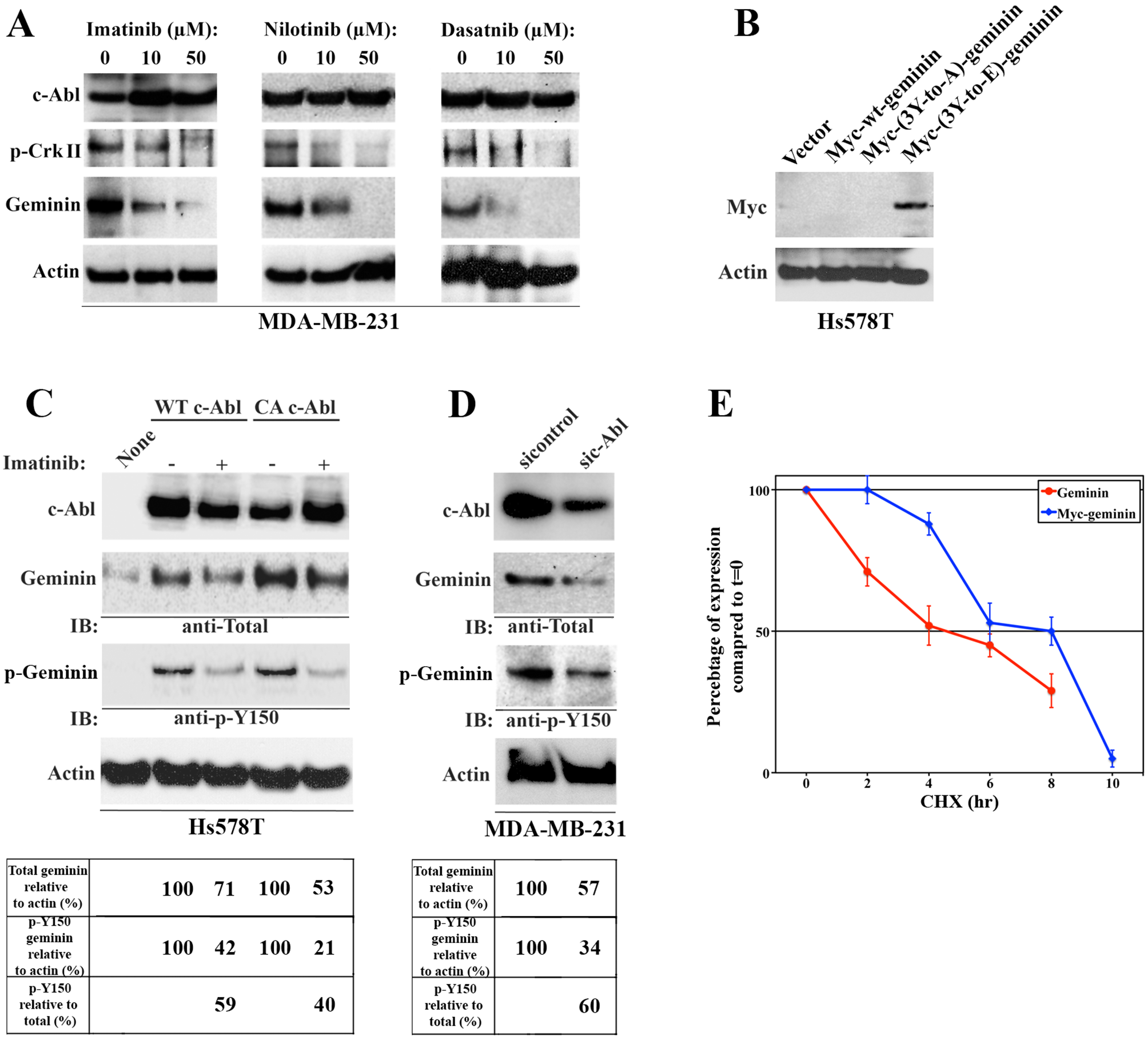

Supplement: Figure S2 — The effect of several c-Abl inhibitors on c-Abl expression and activity, and geminin Y150 phosphorylation by c-Abl, in vivo induces geminin stability. (A) The expression levels c-Abl, p-CrkII and geminin in inducible Gem9 cells treated with 0, 10 and 50 µM of imatinib (left), nilotinib (middle) or dasatinib (right) for 24 h. (B) The expression of Myc-3Y-to-E- and not Myc-WT-geminin or Myc-3Y-to-A-geminin in Hs578T. (C) The re-expression of geminin protein in Hs578T cells reconstituted with WT or CA c-Abl detected using an anti-total geminin or anti-p-Y150 antibodies. Note that imatinib treatment significantly decreased the levels of total and to even higher extent the levels of p-Y150 geminin protein in these reconstituted cells (see Tables below). (D) Expression of total or p-Y150 geminin in MDA-MB-231 cells transfected with si-control or sic-Abl. The level of reduction in total or p-Y150-geminin is presented in Table below the figure. (E) Quantification of the cycloheximide effect on endogenous and exogenous geminin shown in Fig. 4H. (TIF) [file pone.0095663.s002.tif]

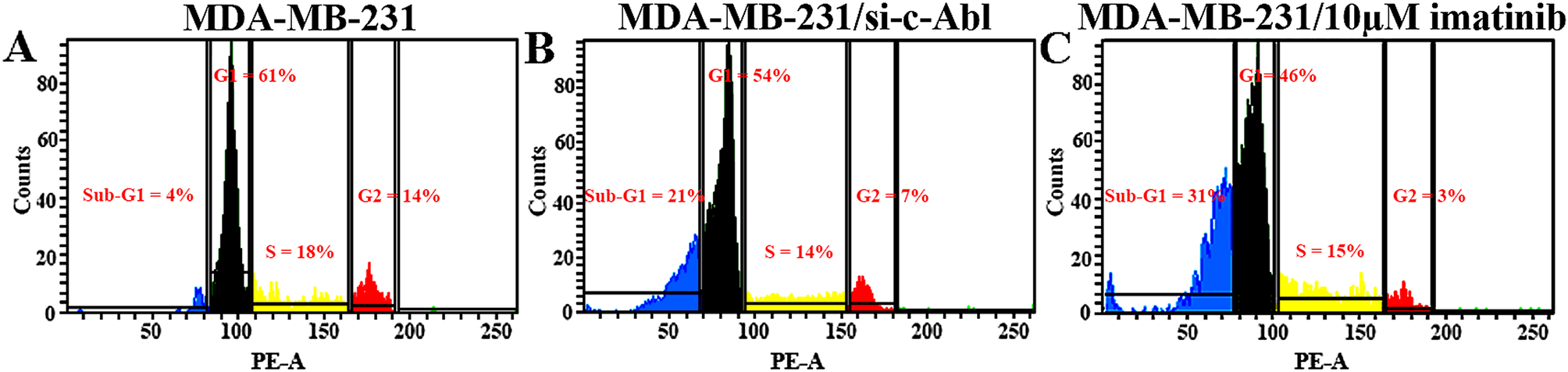

Supplement: Figure S3 — Imatinib kills geminin-overexpressing cells. Representative FACS analysis of MDA-MB-231 cells treated with vehicle (or expressing si-control, A), expressing si-c-Abl (B) or treated with imatinib (C). Note the increased sub-G1 fraction in c-Abl silenced or inactivated cells. (TIF) [file pone.0095663.s003.tif]

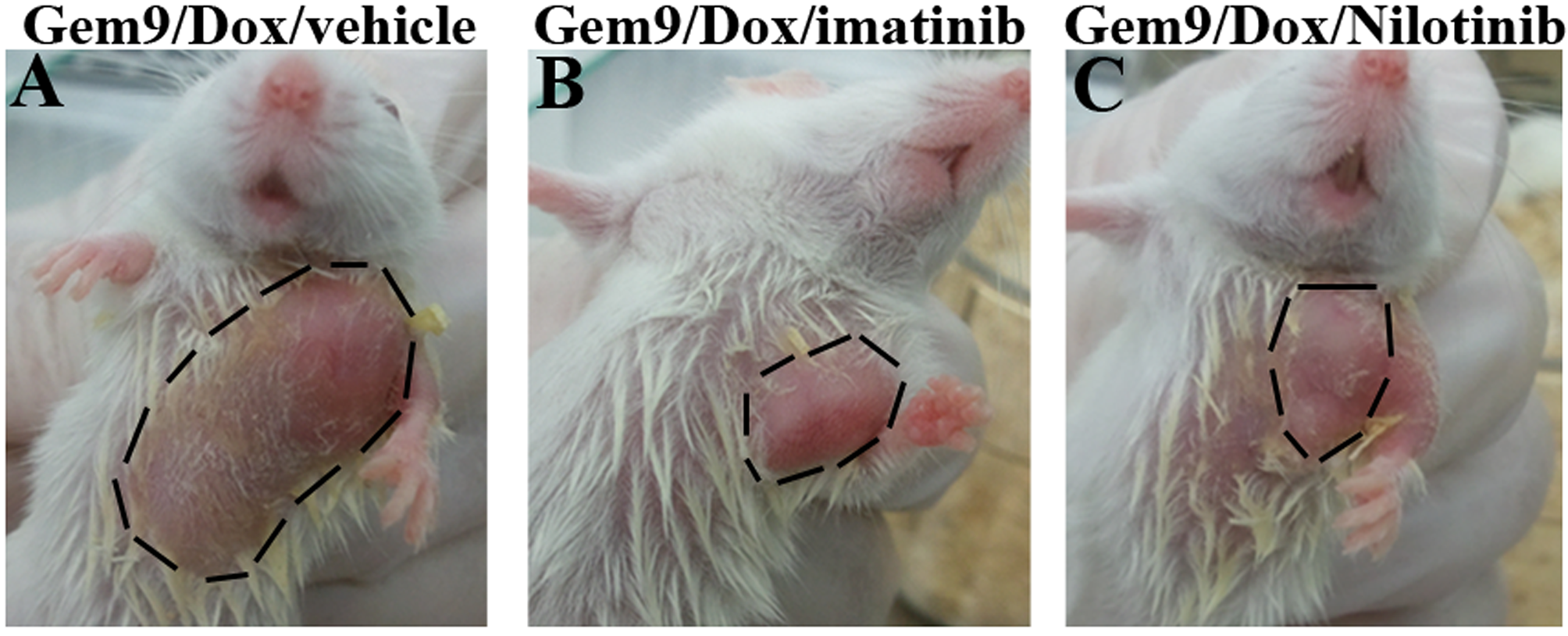

Supplement: Figure S4 — The effect of c-Abl inactivation on geminin overexpressing tumors. Representative images of inducible Gem9 tumors developed in SCID mice following treatment with vehicle (A), imatinib (B) or nilotinib (C). (TIF) [file pone.0095663.s004.tif]
