# Supplementary material for: Geminin Overexpression Promotes Imatinib Sensitive Breast Cancer: A Novel Treatment Approach for Aggressive Breast Cancers, Including a Subset of Triple Negative
Source: PLoS One. 2014 Apr 30;9(4):e95663. doi: 10.1371/journal.pone.0095663 (PMC4005756; doi:10.1371/journal.pone.0095663)
Supplement: Table S1 — The association between geminin overexpression and overexpression of nuclear or cytoplasmic c-Abl in breast tumors. (DOCX) [file pone.0095663.s005.docx]

**Table S1. The association between geminin overexpression and overexpression of nuclear or cytoplasmic c-Abl in breast tumors.**

| **Staining Score** | | **Nuclear c-Abl (n=168)**  **(^a^*p*<0.0001)** | | | **Total (%)** | **Cytoplasmic c-Abl (n=127)**  **(*p*=0.9)** | | | **Total (%)** |
| --- | --- | --- | --- | --- | --- | --- | --- | --- | --- |
|  |  | **0-1**  **(%)** | **2**  **(%)** | **3**  **(%)** |  | **0-1**  **(%)** | **2**  **(%)** | **3**  **(%)** |  |
| **Geminin** | **0-1 (%)** | 1  (0.60) | 1  (0.60) | 1  (0.60) | **3**  **(1.79)** | 41  (32.23) | 56  (44.09) | 30  (23.62) | **127**  **(100)** |
|  | **2 (%)** | 3  (1.79) | 6  (3.57) | 5  (2.98) | **14**  **(8.33)** | 0  (0) | 0  (0) | 0  (0) | **0**  **(0)** |
|  | **3 (%)** | 4  (2.38) | 5  (2.98) | 142 (84.52) | **151**  **(89.88)** | 0  (0) | 0  (0) | 0  (0) | **0**  **(0)** |
| **Total**  **(%)** | | **8**  **(4.76)** | **12**  **(7.14)** | **148**  **(88.10)** | **168**  **(100)** | **41**  **(32.23)** | **56**  **(44.09)** | **30**  **(23.62)** | **127**  **(100)** |

a is Fisher's exact *p*-value
