# Supplementary material for: Geminin Overexpression Promotes Imatinib Sensitive Breast Cancer: A Novel Treatment Approach for Aggressive Breast Cancers, Including a Subset of Triple Negative
Source: PLoS One. 2014 Apr 30;9(4):e95663. doi: 10.1371/journal.pone.0095663 (PMC4005756; doi:10.1371/journal.pone.0095663)
Supplement: Table S2 — Relationships between geminin expression and tumor characteristics in Her2+ and TN/BL breast cancer tumor samples. (DOCX) [file pone.0095663.s006.docx]

**Table S2. Relationships between geminin expression and tumor characteristics in Her2^+^ and TN/BL breast cancer tumor samples.**

|  | **Her2^+^ (n=32)** | | **TN/BL (n=72)** | |
| --- | --- | --- | --- | --- |
|  | **geminin-positive** | **geminin-negative** | **geminin-positive** | **geminin-negative** |
| **Characteristics** | (%) | (%) | (%) | (%) |
|  | (n=21) | (n=11) | (n=46) | (n=26) |
| **Tumor Grade**  **(as modified nuclear grade)** |  |  |  |  |
| **1** | 0 (0) | 0 (0) | 0 (0) | 0 (0) |
| **2** | 5 (25) | 12 (100) | 13 (28) | 21 (81) |
| **3** | 16 (75) | 0 (0) | 33 (72) | 5 (19) |
| ***^a^p-value*** | **0.0012** | **0.0723** | **0.0056** | **0.0525** |
| **Tumor Stage** |  |  |  |  |
| *In situ* | 0 (0) | 0 (0) | 0 (0) | 0 (0) |
| Localized | 8 (40) | 7 (67) | 18 (39) | 16 (62) |
| Lymph-node | 4 (20) | 4 (33) | 15 (33) | 10 (38) |
| Distant Mets | 9 (40) | 0 (0) | 13 (28) | 0 (0) |
| ***p-value*** | **0.0109** | **0.0503** | **0.0022** | **0.0042** |

^a^To compare multiple groups with one control group, analysis of variance (ANOVA) was used. *p*-values (two-sided) <0.05 were considered statistically significant
